# Pesticides and habitat loss additively reduce wild bees in crop fields

**DOI:** 10.1038/s41559-025-02924-z

**Published:** 2025-12-10

**Authors:** Anina Knauer, Subodh Adhikari, Georg K. S. Andersson, Emilie Andrieu, András Báldi, Péter Batáry, Jordi Bosch, Sara L. Bushmann, Domingo Cano, Romain Carrié, Bryan N. Danforth, Francis A. Drummond, Diane Esquerré, Daniel García, Claudio Gratton, Peter A. Hambäck, Anne-Kathrin Happe, Veronica Hederström, Andrea Holzschuh, Philippe Jeanneret, Riina Kaasik, Temitope Kehinde, Jessica Knapp, Anikó Kovács-Hostyánszki, Claire Kremen, Ilona Leyer, Gisela Lüscher, Rachel Mallinger, Riho Marja, Carlos Martínez-Núñez, Fabian D. Menalled, Leithen K. M’Gonigle, Marcos Miñarro, Anne-Christine Mupepele, Charlie C. Nicholson, Mark Otieno, Annie Ouin, Mia G. Park, Maria-Helena Pereira-Peixoto, Antonio J. Pérez, Simon G. Potts, Annette Reineke, Pedro J. Rey, Taylor H. Ricketts, Justine Rivers-Moore, Stuart Roberts, Laura Roquer-Beni, Maj Rundlöf, Ulrika Samnegård, Michael J. Samways, Janine M. Schwarz, Oliver Schweiger, Henrik G. Smith, Ingolf Steffan-Dewenter, Louis Sutter, Giovanni Tamburini, Deniz Uzman, Eve Veromann, Aude Vialatte, Eneli Viik, Mark J. F. Brown, Alexandra-Maria Klein, Matthias Albrecht

**Affiliations:** 1 Agroecology and Environment, Agroscope, Zurich, Switzerland; 2 Department of Biology, Utah State University, Logan, UT USA; 3 Department of Biology, Lund University, Lund, Sweden; 4 DYNAFOR, University of Toulouse, INRAE, Castanet-Tolosan, France; 5 LTSER Zone Atelier PYRÉNÉES GARONNE, Auzeville-Tolosane, France; 6 “Lendület” Landscape and Conservation Ecology, MTA-HUN-REN Centre for Ecological Research, Vácrátót, Hungary; 7 “Lendület” Landscape and Conservation Ecology, HUN-REN Centre for Ecological Research, Vácrátót, Hungary; 8 Department of Agriculture, Ecotrophology, and Landscape Development, Anhalt University of Applied Sciences, Bernburg, Germany; 9 CREAF - Ecology Unit, Universitat Autònoma de Barcelona, Bellaterra, Spain; 10 George Stevens Academy, Blue Hill, ME USA; 11 School of Biology and Ecology, University of Maine, Orono, ME USA; 12 Departamento de Biología Animal, Biología Vegetal y Ecología, University of Jaén, Jaén, Spain; 13 Centre for Environmental and Climate Science, Lund University, Lund, Sweden; 14 Department of Entomology, Cornell University, Ithaca, NY USA; 15 Biodiversity Research Institute, University of Oviedo, Oviedo, Spain; 16 Department of Entomology, University of Wisconsin–Madison, Madison, WI USA; 17 Department of Ecology, Environment & Plant Sciences, Stockholm University, Stockholm, Sweden; 18 Agroecology, University of Göttingen, Göttingen, Germany; 19 Department of Animal Ecology and Tropical Biology, University of Würzburg, Würzburg, Germany; 20 Estonian University of Life Sciences, Tartu, Estonia; 21 Obafemi Awolowo University, Ile-Ife, Nigeria; 22 Institute for Resources, Environment & Sustainability, University of British Columbia, Vancouver, British Columbia Canada; 23 Department of Applied Ecology, Geisenheim University, Geisenheim, Germany; 24 Department of Ecology and Evolution, Estación Biológica de Doñana EBD (CSIC), Seville, Spain; 25 Department of Land Resources and Environmental Sciences, Montana State University, Bozeman, MT USA; 26 Department of Environmental Sciences, Policy and Management, University of California, Berkeley, CA USA; 27 Department of Biological Sciences, Simon Fraser University, Burnaby, British Columbia Canada; 28 Servicio Regional de Investigación y Desarrollo Agroalimentario (SERIDA), Villaviciosa, Spain; 29 Department of Nature Conservation and Landscape Ecology, University of Freiburg, Freiburg, Germany; 30 Gund Institute for Environment, University of Vermont, Burlington, VT USA; 31 Department of Biology, Marine Biology, and Environmental Science, Roger Williams University, Bristol, RI USA; 32 Department of Water and Agricultural Resource Management, University of Embu, Embu, Kenya; 33 Chair of Nature Conservation and Landscape Ecology, University of Freiburg, Freiburg, Germany; 34 Centre for Agri-Environmental Research, University of Reading, Reading, UK; 35 Department of Crop Protection, Geisenheim University, Geisenheim, Germany; 36 Instituto Interuniversitario de Investigación del Sistema Tierra de Andalucía, University of Jaén, Jaén, Spain; 37 Department of Conservation Ecology and Entomology, Stellenbosch University, Stellenbosch, South Africa; 38 Department of Community Ecology, Helmholtz Centre for Environmental Research UFZ, Halle, Germany; 39 German Centre for Integrative Biodiversity Research (iDiv) Halle-Jena-Leipzig, Leipzig, Germany; 40 Department of Soil, Plant and Food Sciences, University of Bari, Bari, Italy; 41 Centre of Estonian Rural Research and Knowledge (METK), Jõgeva, Estonia; 42 Department of Biological Sciences, Royal Holloway University of London, Egham, UK

**Keywords:** Agroecology, Community ecology, Biodiversity, Conservation biology, Ecosystem services

## Abstract

Pesticide use and habitat loss are major anthropogenic drivers of bee decline, raising global concerns about impaired crop pollination. However, the relative importance of these stressors and their combined impact on bee assemblages comprising species with different traits, such as body size or nesting strategy, remains unknown. Here we addressed these key knowledge gaps in a global quantitative synthesis analysing bee assemblage data from 681 crop fields across three continents. We found that both local pesticide hazards and decreasing proportions of semi-natural habitats in surrounding landscapes negatively affected wild bee abundance and species richness in crop fields, while pesticides additionally reduced functional and phylogenetic diversity. Semi-natural habitat availability did not buffer against these negative pesticide effects, nor did we identify any specific traits rending bees more vulnerable to one of the two drivers. Our findings highlight the pressing need to reduce non-target effects of pesticide use and emphasize that conservation and restoration of semi-natural habitats successfully promote wild bees, but are insufficient strategies to mitigate pesticide-driven losses of wild bee pollinators from crop fields.

## Abstract (web summary)

The authors synthesize bee assemblage data from 681 crop fields across three continents, finding that local pesticide hazards and decreasing adjacent semi-natural habitats both negatively affected wild bee abundance and species richness in crop fields, while pesticides also reduced functional diversity.

## Main

The abundance and functional diversity of wild bees play a key role for plant pollination, but their declines in many regions of the world put wider biodiversity, the functioning of terrestrial ecosystems and pollination services to crops at risk^1–3^. A major driver of this decline is the loss of suitable habitats through agricultural intensification^4,5^. In addition, this intensification is accompanied by increased use of agrochemicals, many of which pose a threat to pollinators in agroecosystems^6–9^. Sustainable agricultural practices and stable long-term yields of insect-pollinated crops require the conservation of several facets of pollinator community structure^1,10,11^, but it remains unknown how the use of pesticides affect wild bees in different cropping systems worldwide with respect to their abundance, species richness, functional and phylogenetic diversity. Moreover, we currently lack a general understanding of how additional stressors, such as the loss of semi-natural habitats (SNH) in landscapes surrounding crop fields, may accelerate the decline of wild bees through synergistic interactions^12^.

**Extended Data Fig. 1:**
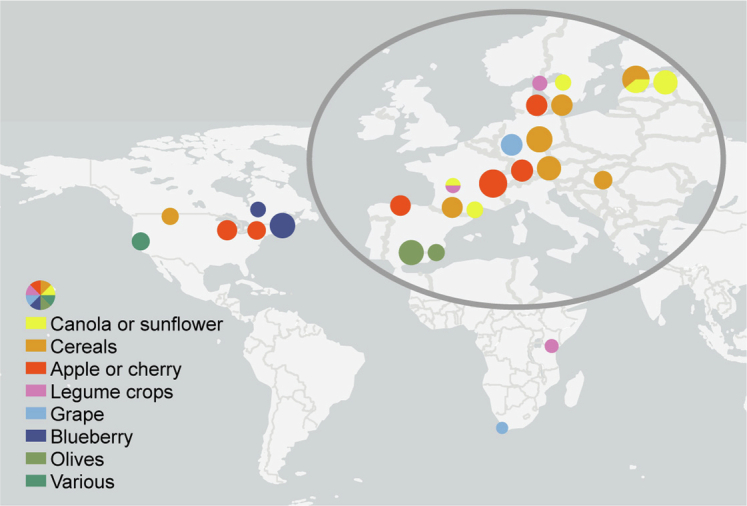
Worldwide distribution of study sites (N = 681 sites) and crop type with a closer view of Europe (ellipse). Circle sizes are proportional to the number of sites per region.

Here we address these research gaps through a global analysis of 36 primary datasets covering 681 agricultural fields across various cropping systems on the African, European and North American continents (Extended Data Fig. 1 and Supplementary Table 1). Datasets were selected on the basis of a systematic literature search of published field studies on the effects of pesticide use and SNH loss on wild bee assemblages in crop fields. The analysed data includes information on the abundance and potential response traits (body size, lecty, sociality, nest location, nesting strategy and kleptoparasitism; Extended Data Table 1) of 910 bee species (19,593 specimens). Two measurements of pesticide hazard in focal fields were used: (1) high versus low intensity of pesticide use based on production systems (conventional or organic, additionally supported by information on typical pesticide management for example through farmer interviews), available for 27 datasets, or (2) hazard quotients (HQ) that incorporate application rates and the toxicity of applied pesticides (including insecticides, fungicides and herbicides) to bees^13^, available for 28 datasets with a total of 6,667 individual pesticide applications. Studies selected sites to achieve representative gradients of SNH proportions in landscapes and/or pesticide hazard, or site selection was random in relation to the respective driver. Approximately half the crops grown in focal fields were attractive to bees and the proportion of SNH (for example, semi-natural grasslands, forests, shrublands and hedgerows; Supplementary Table 2) in landscapes ranged typically from 0 to 80%. Comparison to continental databases and literature corroborates that these ranges can be considered representative for these global growing regions^14–16^.

## Results

### Effects on bee communities in crop fields

As individual bees are lost from communities because of exposure to anthropogenic stressors, abundance is decreased and species are subsequently lost through random attrition, with potential consequences for functional and phylogenetic diversity^17^. In addition, anthropogenic stressors are hypothesized to affect bee species differently depending on how the traits of a species shape their response to pesticide hazard and loss of SNH^2,18^. Consequently, stressors could restructure communities by favouring species with trait combinations that help them persist in simplified and intensively managed agroecosystems, while resulting in population decline and even extinction of species with less favourable traits^19^. The resulting bee assemblages would be characterized by an altered community structure with expected effects on functional mean pairwise distance (MPD), evenness or specialization. Beyond these parameters, we also measured phylogenetic MPD to test for environmental filtering by unidentified traits associated with phylogenetic relatedness. This metric is expected to decrease with increasing stressor intensity, because traits that enable species to cope better with stressors (for example, detoxification enzymes) are often conserved within taxonomic groups^2,20^.

**Fig. 1:**
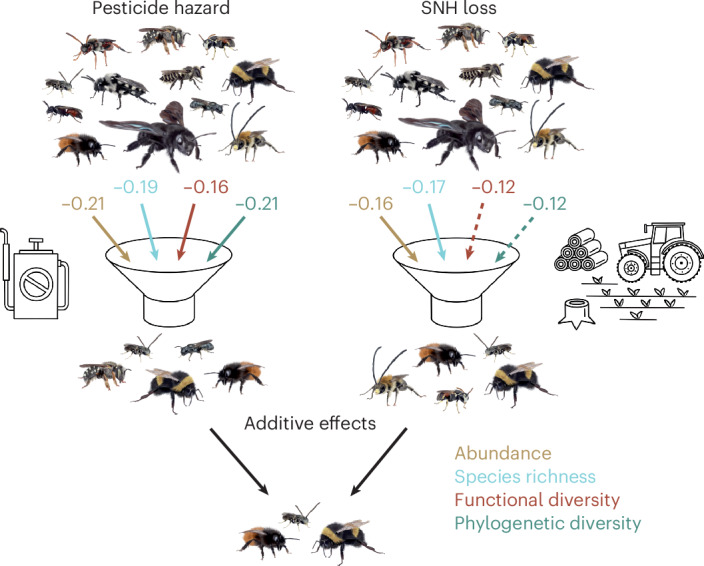
Loss of SNH in the agricultural landscape and pesticide hazard additively reduced bee abundance and diversity in crop fields. A total of 681 crop fields were sampled. Pesticide hazard was calculated as an HQ based on pesticide application protocols considering application rates and the toxicity of active ingredients to bees (LD_50_). Numbers represent standardized slope estimates of linear mixed effects models accounting for non-independence within dataset, solid lines indicate significant effects (*P* ≤ 0.05) and dashed lines trends (0.05 < *P* ≤ 0.1). Credit: Illustrations by Janine Schwarz; bee photos from Apidarium (https://apidarium.de).

**Fig. 2:**
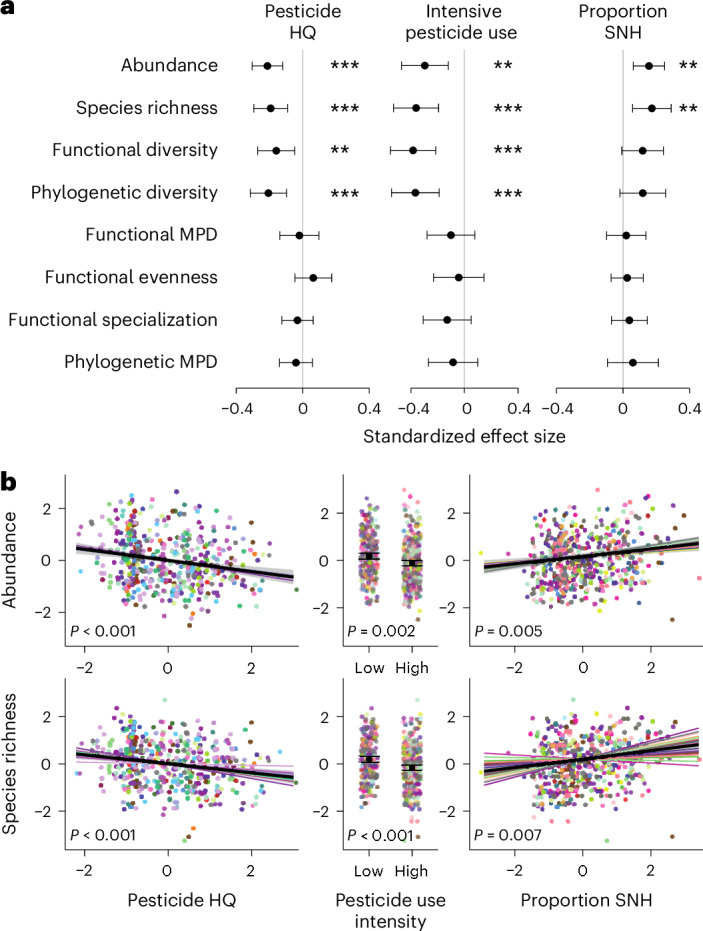
Effects of pesticide hazard and SNH amount on wild bee community descriptors. **a**,**b**, Estimates ±95% confidence intervals (CI) derived from linear mixed effects models accounting for non-independence within dataset (*n* = 681 sites) on the effect of the pesticide HQ (calculated from pesticide application protocols considering application rates and the toxicity of active ingredients to bees) (left), high pesticide-use intensity (based on production system considering typical application protocols) (middle) and the proportion of SNH (right) in surrounding landscapes on bee assemblage metrics. **a**, Effects on abundance, species richness and different metrics of functional and phylogenetic diversity (MPD = mean pairwise difference). Stars indicate significance levels (**P < 0.01; ***P < 0.001). **b**, Effects on bee abundance and species richness with colours indicating datasets and corresponding random slopes. Source data

**Extended Data Fig. 2:**
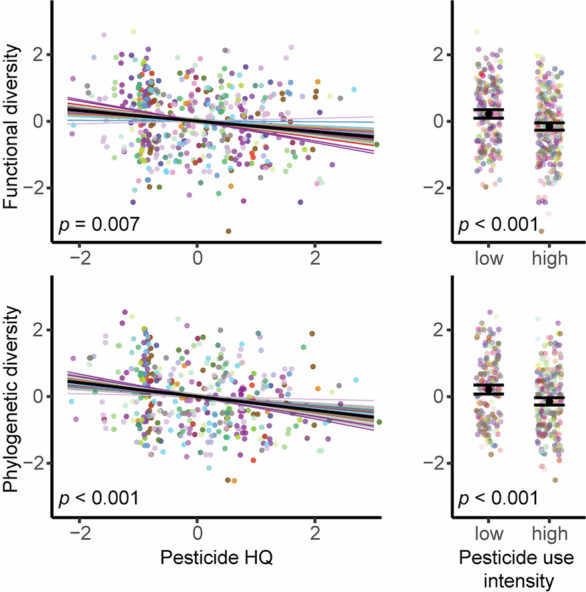
Effects of pesticide hazard on bee communities. Estimates ± 95% CI derived from linear mixed effects models accounting for non-independence within dataset (N = 681 sites) on the effect of the pesticide hazard quotient (HQ, calculated from pesticide application protocols considering application rates and the toxicity of active ingredients to bees) (left) and high pesticide-use intensity (based on production system considering typical application protocols) (right) on functional and phylogenetic diversity of bees in crop fields (from top down). Colors indicate datasets and corresponding random slopes.

We found that bee abundance and species richness decline with decreasing proportions of SNH and increasing pesticide hazard in agricultural landscapes. Pesticide hazard was additionally associated with lower functional and phylogenetic diversity of wild bee assemblages in crop fields (Figs. 1 and 2and Extended Data Fig. 2). These relationships were detected irrespective of whether hazard was quantified on the basis of pesticide-use intensity or as HQ. Additionally, these effects did not vary significantly across the major global growing regions studied (North America, Europe and Africa). Furthermore, we did not detect shifts in the distribution of traits within bee assemblages along gradients of increasing pesticide hazard or decreasing proportions of SNH in landscapes as the functional and phylogenetic MPDs and the functional evenness and specialization of communities were not related to these stressors (Fig. 2). In accordance with ref. ^21^, these results suggest that the community disassembly of bees observed in relation to pesticides and SNH loss is not strongly driven by specific traits.

The lack of general patterns of community filtering may arise when the fitness consequences of certain traits or trait combinations depend on the focal crop and/or the landscape context^22^. For example, pesticide exposure of ground-nesting bees through contaminated soil may occur mainly in crops with low vegetation cover as bees depend on bare soil to build their nests, whereas exposure may be much lower in dense crops^23^. Pesticide exposure may also depend on the foraging preference of bees for crop flowers or other floral resources in crop fields^18,24,25^. However, no information was available on specific foraging preferences beyond the degree of floral specialization. At the landscape level, the presence and composition of different types of SNH may shape the availability of specific floral resources and nesting sites for ground-nesting versus above-ground-nesting bees leading to undirected patterns of trait filtering by the proportion of SNH.

### Underlying pathways of pesticide effects

**Extended Data Fig. 3:**
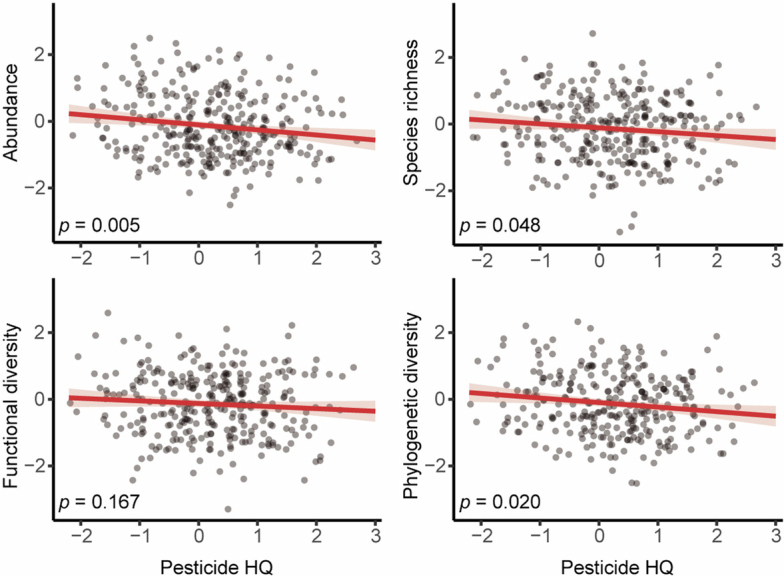
Effects of pesticide hazard quotient (HQ) on bee communities in crop fields within conventional production system. Estimates ± 95% CI derived from linear mixed effects models accounting for non-independence within dataset (N = 322 sites) on the effect of the pesticide hazard quotient (HQ, calculated from pesticide application protocols considering application rates and the toxicity of active ingredients to bees) on abundance, species richness, and functional and phylogenetic diversity of bees in crop fields.

In agricultural systems, there are two main pathways through which the use of pesticides can affect bees: (1) through toxic effects or increasing susceptibility to other stressors when bees are exposed to pesticides via contaminated food or spray contact and (2) through reduced floral resource availability as a result of chemical weed control. While the adverse effects of pesticide-use intensity (based on organic versus conventional production system) cover both pathways, the negative effect of the HQ corroborates direct toxicity as a main route by which bees in crop fields are affected by pesticides (Fig. 2). To confirm that these effects were not mainly driven by underlying differences between production systems, for example in floral resource availability, we additionally tested HQ effects only on the subset of conventionally managed crop fields (322 of the total of 466 fields used in the main analysis across both production systems). Results were consistent and qualitatively similar to analyses including fields from both production systems, showing negative effects on bee abundance, species richness and phylogenetic (but not functional) diversity in conventionally managed fields as HQ increased (Extended Data Fig. 3). These results suggest that bees are harmed by the direct toxic effects of pesticides, which does not exclude any possible additional differences between organic and conventional production systems that contribute to overarching patterns including reduced functional diversity of bees.

**Extended Data Fig. 4:**
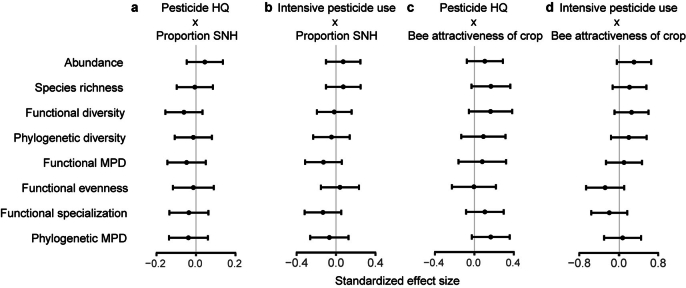
Interaction terms of statistical models used to test for buffering of pesticide hazard by semi-natural habitat (SNH) proportion in landscapes and for increased effects of pesticide hazard in bee-attractive crops. **a**–**d**, Estimates ± 95% CI derived from linear mixed effects models accounting for non-independence within dataset (N = 681 sites) on the studied descriptors of bee communities in crop fields (MPD = mean pairwise distance) by pesticide hazard quotient (HQ, calculated from pesticide application protocols considering application rates and the toxicity of active ingredients to bees) × SNH (**a**), high pesticide use intensity intensity (based on production system considering typical application protocols) × SNH (**b**), HQ × bee attractiveness of focal crop (**c**), high pesticide use intensity × bee attractiveness of focal crop (**d**). Source data

We assumed that the effects of pesticide hazard would be greater in bee-attractive crops during flowering compared with non-attractive crops. In contrast to our predictions, pesticides did not have a stronger negative effect on bee abundance and diversity in bee-attractive crops versus non-attractive crops (Extended Data Fig. 4). This result supports previous evidence that the effects of pesticides can vary substantially across different types of bee-attractive crops^9,26^, and that bees are exposed to pesticides through a variety of routes in agricultural systems. For example, pesticide drift from spray applications or leaching through the soil can result in residues in weeds within fields or in non-target wildflowers along field margins, representing likely routes of oral exposure^27–31^.

A high proportion of SNH could mitigate the negative effects of pesticide use on bees in focal fields through at least three, non-exclusive pathways: (1) through reduced pesticide exposure of bees when also foraging or nesting in SNH where flowers and soils are less contaminated with pesticides compared with those in crop fields, (2) through improved resilience of bees against negative pesticide impacts due to nutritional benefits obtained from floral resources in SNH and (3) through reduced mortality rates at the population level in landscapes with larger and more distributed populations due to larger amounts of SNH (that is, when SNH provides valuable habitat, a smaller proportion of the population forages in crops and is exposed to pesticides)^26,32–34^. Landscape configuration may further modulate such mitigation effects as better connected SNH patches and crop fields could facilitate the use of complementary floral resources by bees and improve recolonization of crop fields from SNH. However, we found that impacts of pesticide hazards and SNH availability on bee assemblages were additive, not interactive, indicating that variation in bee abundance and diversity related to pesticide use was not mitigated by increasing proportions of SNH in the vicinity of crops (Extended Data Fig. 4). This result was consistent across local field sizes and landscape configurations (edge density calculated on the basis of the global maps of land use/land cover (LULC) derived from ESA Sentinel-2 imagery (Methods)) (no significant three-way interactions).

Buffering effects of flower availability on the impact of pesticides on bees have been documented in individual studies^9,13,35,36^. However, our study suggests that this may not be a general pattern, although between-study heterogeneities in landscape-wide flower availability may have obscured buffering in certain agroecosystems. Furthermore, it is conceivable that the high density of floral resources in agricultural fields is generally so attractive to bees that they forage on these flowers until they reach harmful doses of pesticides, even if they occasionally also forage in SNH^37^. Additionally, foraging on wildflowers in SNH could be impaired by sublethal pesticide effects that reduce cognitive abilities and memory^38^ or the potential for buffering may be limited by spray drift contaminating wildflowers and nesting sites in SNH^27,28^. Irrespective of the exact mechanism, our study underpins the importance of conserving and restoring SNH for maintaining bee abundance and diversity in crop fields^1,2,4^, but at the same time cautions that such habitats may have limited potential to mitigate pesticide hazards for pollinators^39^.

### Effects on beta diversity of bees

Anthropogenic stressors can also shape patterns of species composition across agroecosystems with different levels of intensification (reflected in beta diversity), with different consequences for the overall species pool in the region (that is, gamma diversity)^12^. Nestedness reflects a disassembly process characterized by pruning species from the species pool resulting in a subset of species, whereas turnover indicates a species loss accompanied by simultaneous dissimilarity in species composition resulting from the replacement of a subset of the species pool (Supplementary Fig. 1). While turnover among sites may mitigate patterns of bee decline in a region to some extent, nestedness inevitably reduces regional diversity^17^. A better mechanistic understanding of how pesticide use and SNH loss in agroecosystems shape the several components of community disassembly is, therefore, relevant to protecting bees.

**Fig. 3:**
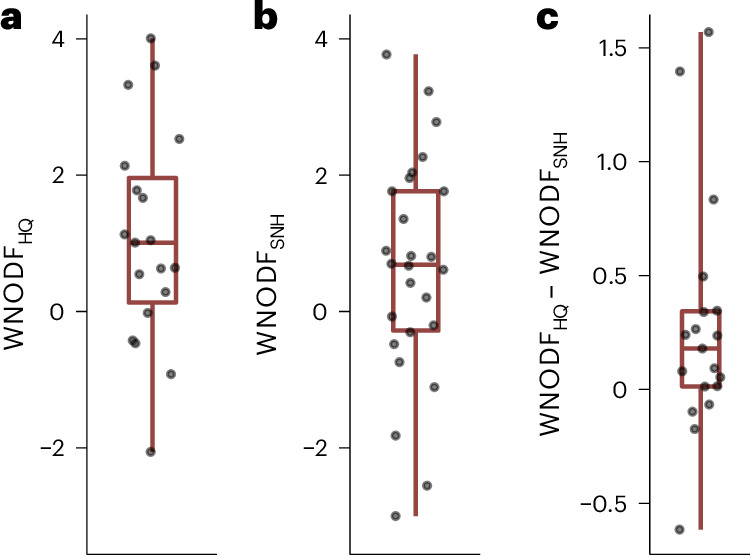
Nestedness of bee communities across sites of increasing pesticide hazards versus decreasing proportions of SNH. **a**, WNODF values along increasing HQ were significantly larger than zero (two-sided one-sample *t*-test: mean WNODF = 1.08, 95% CI = 0.31–1.84; *t* = 2.96, *n* = 19, *P* = 0.008). **b**, WNODF values along decreasing SNH proportions were not significantly different from zero (two-sided one-sample *t*-test: mean WNODF = 0.61, 95% CI = −0.06–1.27; *t* = 1.86, *n* = 26; *P* = 0.074). **c**, Pesticide hazard generally contributed more to nestedness than SNH loss when the two WNODF values obtained per study were compared (two-sided paired Wilcoxon test: *n* = 19 pairs, *P* = 0.014). WNODF is calculated across gradients of several locations. Therefore, each data point represents a nestedness measure from a single study. Boxplots show the median (line), interquartile range (box; 25th–75th percentiles) and range (whiskers).

**Extended Data Fig. 5:**
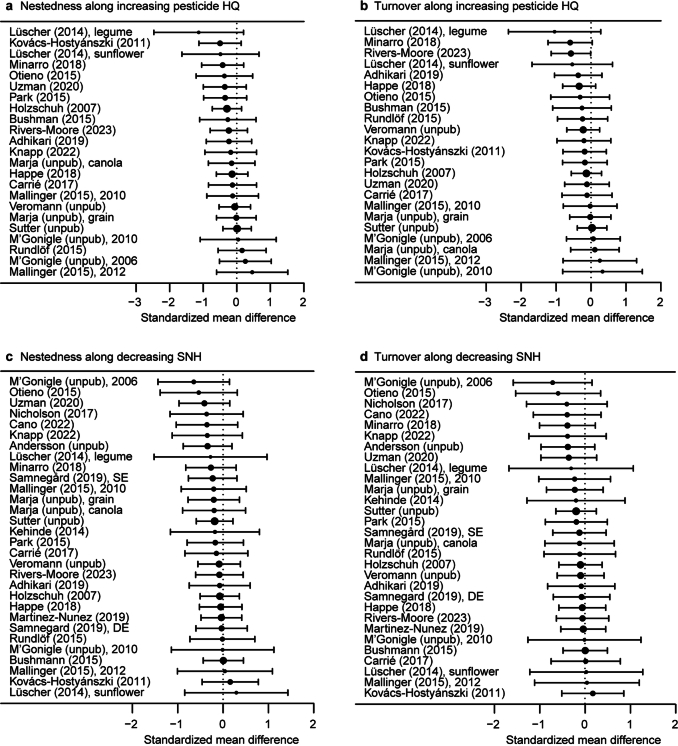
Nestedness and turnover of bee communities along gradients of stressor intensity. (**a**) Nestedness along increasing pesticide hazard (hazard quotient HQ) (standardized mean differences = -0.17, 95% CI = -0.32 to -0.03, N = 23, t = -2.51, p = 0.020). (**b**) Turnover along increasing pesticide hazard (HQ) (standardized mean differences = -0.19, 95% CI = -0.33 to -0.05, N = 23, t = -2.77, p = 0.011). (**c**) Nestedness along decreasing proportions of semi-natural habitat (SNH) in surrounding landscapes (standardized mean differences = -0.16, 95% CI = -0.27 to -0.04, N = 30, t = -2.75, p = 0.010). (**d**) Turnover along decreasing proportions of SNH in surrounding landscapes (standardized mean differences = -0.15, 95% CI = -0.26 to -0.03, N = 30, t = -2.62, p = 0.014). Shown are standardised mean differences between the losing and gaining component of nestedness or turnover ± 95% CI per study from a two-sided meta-analysis model. A negative estimate indicates a loss of species along gradients of increasing intensity of stressors and is obtained when the losing component is larger than the gaining component. Point sizes reflect sample sizes per study and their association with the weighting in the model.

Bee assemblages showed stronger nestedness than would be expected by chance along gradients of increasing pesticide hazard, but in contrast, effects were weak and not significant along gradients of decreasing SNH (nestedness measured as WNODF, a metric based on weighted overlap and decreasing fill considering species abundances; Methods) (Fig. 3a,b). When directly comparing pairs of nestedness responses obtained from the same studies, nestedness was greater along crop fields of increasing pesticide hazard compared with decreasing SNH (Fig. 3c). However, when studying beta diversity based on species occurrence data, both nestedness and turnover characterized patterns of species variation among crop fields related to pesticide hazard and SNH availability (Extended Data Fig. 5). These findings are in line with those for alpha diversity and indicate that high pesticide hazards adversely affect different key properties of bee diversity within and across crop fields.

## Discussion

Although habitat loss has received more attention than other factors as a cause of bee diversity decline in agroecosystems^2,11,40,41^, our findings suggest that, in addition to the clear effects of SNH, local pesticide hazard is associated with lower wild bee abundance and diversity in crop fields. Pesticide hazard also related more to the pruning of bees from assemblages found across different crop fields than the difference in SNH between landscapes. However, it is important to note that, while we quantified impacts of pesticide hazard and SNH loss on wild bee assemblages visiting crop fields in this synthesis, the loss of SNH may have even more pronounced impacts on bee abundance and diversity within those habitats themselves^42^.

This study demonstrates that the ability of SNH to provide a buffer against the negative impacts of pesticides on bee communities is not ubiquitous. We posit that the relative quality of SNH, such as floral and nesting resource availability, may be as—if not more—important than SNH quantity or configuration. Yet, while not all types of SNH necessarily represent good foraging or nesting habitats for bees^43,44^, cropland can provide valuable resources for certain bee species, especially when combined with other resources that bees depend on in heterogeneous agricultural landscapes^45,46^. To further understand the roles of SNH and to develop landscape management strategies to mitigate adverse pesticide effects, future work should consider the quality of different SNH habitat types with respect to their nest site availability and the distribution, seasonality and quality of floral resources—beyond SNH amount and configuration.

Our findings that pesticide hazard was associated with low wild bee abundance, species richness and functional and phylogenetic diversity suggest that current pesticide regulations are insufficient to prevent the loss of wild bee pollinators in crop fields and thus raise concerns about the sustainability of intensive crop production systems relying on high pesticide inputs. This is particularly true for crops that are dependent on pollinators, as taxonomically and functionally diverse pollinator communities are important for pollination services^1,2,47,48^. Furthermore, the loss of functional diversity may constrain community resistance and resilience to future environmental stress^12^. The situation may be even more problematic in regions such as South America or Asia, which have the highest pesticide use per agricultural land area globally, still including highly bee-hazardous pesticides such as neonicotinoids^49,50^, but data from such regions were lacking for our analysis. Therefore, to safeguard bees and other pollinators and to maintain pollination services to crops and wild plants, coordinated strategies are required to achieve both reduction of pesticide risks for bees and promotion of their habitats in agricultural landscapes.

Beyond strengthened pesticide regulation and technological advances, integrated pest and pollinator management^51^ may contribute to reducing pesticide hazards to bees and other pollinators. Moreover, holistic agroecological approaches, which integrate co-creation processes among stakeholders to develop more sustainable, resilient and diversified production systems, provide promising transition paths towards a less pesticide-input dependent agriculture^52^. We therefore encourage the consistent implementation and reinforcement of global policy efforts such as the COP 15 goals on the Convention on Biological Diversity and the European Farm to Fork strategy^53,54^.

## Methods

We followed the PRISMA extension for ecology and evolution^55^ to fulfil quality standards for data collection, analysis and reporting throughout the research process. A summary of how these recommendations were adapted to our analytical approach of a quantitative synthesis can be found in Supplementary Table 3.

### Data collection

A systematic Web of Science search (core collection database) using ‘bee’ AND (‘wild bee abundance’ OR ‘diversity’ OR ‘species richness’) AND (‘organic’ OR ‘production system’ OR ‘pesticides’ OR ‘agrochemicals’ OR ‘insecticides’ OR ‘fungicides’) was performed in June 2019 to find a representative sample of studies. The search yielded a total of 170 publications, which were checked for eligibility on the basis of the following criteria: (1) the studies were entirely observational, with no manipulation of pesticide exposure; (2) the studies characterized wild bee communities in crop fields and/or their margins; (3) information on field-realistic pesticide use was collected for the focal crop field where bees were captured or for crop fields adjacent to field margins in which bees were collected; (4) the proportion of SNH in agricultural landscapes surrounding the local field was measured; (5) the studies used a paired design with high and low pesticide use in landscapes of similar proportion of SNH or sites were selected along independent gradients of pesticide use and proportion of SNH; and (6) studies identified bees to species (or morphospecies) level and had a sufficient sampling effort (more than ten bees on average per sampling site) as required for the quantification of species richness and functional diversity. Corresponding authors of suitable studies were asked to share their data and, to minimize potential publication bias and to maximize the number of relevant datasets, we asked them for further potentially suitable unpublished datasets collected by themselves or researchers in their network (Supplementary Fig. 2).

This search resulted in 26 studies and 36 datasets, including data from 681 sites, mostly from Europe and North America (Supplementary Fig. 2, Supplementary Table 1 and Extended Data Fig. 1), which were collected between 2003 and 2018. We defined a dataset as data collected by the same group of researchers for a particular crop species across a replicated set of different study sites in the same time period^11,56^. If data were collected across several years, data collected in different years were considered as separate datasets as long as different sites were studied across years (Supplementary Table 1). Of these 36 datasets, 28 (from 19 studies, 466 sites) contained detailed information on pesticide application protocols during the years of bee collection and 27 datasets (from 20 studies, 514 sites) contained information on low versus high pesticide-use intensity based on differences in the production systems (conventional or organic, additionally supported by information on typical pesticide management, for example, through farmer interviews) (Supplementary Table 2). In all subsequent analyses, these two types of datasets were analysed separately (see section on ‘Statistical analysis’ below).

### Bee assemblage data

Bee assemblages were sampled in focal crop fields and/or along field margins with different sampling methods, mainly including timed observations and pan trapping (Supplementary Table 2). On the basis of the raw data, we calculated a range of wild bee assemblage metrics for each site, including abundance and measures of taxonomic, functional and phylogenetic diversity. *Apis mellifera* was excluded from all metrics because its abundance strongly depends on management, although it can also be affected by stressors. While species richness and functional and phylogenetic diversity are expected to decline along with a general loss of bees through random attrition^17,57^, functional evenness, functional specialization and functional and phylogenetic MPD should only change when community composition is altered as a result of environmental filtering^2,10,57–60^. Functional evenness of an assemblage expresses the weighted regularity of species in the functional space (along the minimum spanning tree) while functional specialization represents the weighted mean distance of the species in an assemblage to the centroid of the global species pool (that is, centre of the functional space). Functional and phylogenetic MPD are the mean pairwise distance between all pairs of species found in an assemblage in the functional space or along the phylogenetic tree, respectively^58,61,62^. Total functional and phylogenetic diversity per site were measured as the total branch length of the functional and phylogenetic dendrogram^63,64^.

Functional and phylogenetic diversity were calculated with the alpha function from the BAT package^65^ in R v.4.3.2^66^. Abundance weighted functional MPD, evenness and specialization were calculated with the mFD package^58^, while phylogenetic MPD was obtained from the Picante package^67^. We used abundance weighted measures rather than species occurrence data as the former are more sensitive to changes in community composition and environmental filtering. However, analyses using metrics from species occurrence data yielded qualitatively similar results. Since functional traits can be correlated, we calculated functional community descriptors using independent principal components obtained from a principal coordinates analysis (PCoA) using the Gower multitrait dissimilarity matrix obtained with the gawdis package^68^. Gower multitrait dissimilarity matrices were calculated separately for each dataset to optimize the quality of the PCoA-based functional spaces for each set of comparable bee communities^58^. For phylogenetic diversity, we used Grafen branch lengths based on taxonomic relationships of species^10^.

For estimates of functional diversity, we included traits of 910 wild bee species and morphospecies (19,593 specimens) from six families (Andrenidae, Apidae, Colletidae, Halictidae, Megachilidae and Melittidae). We used traits that have been considered to modulate pesticide exposure and susceptibility to habitat loss^2,18,20,69–75^; that is, body size, lecty, sociality, nest location, nesting strategy and kleptoparasitism (Extended Data Table 1).

Trait data were partly provided in the primary datasets, but were supplemented with literature and existing databases such as the Palaearctic Osmiine Bees database^76^. For body size and lecty we obtained trait information for 76% and 72% of species, respectively (data mostly missing for morphospecies), while missing values for the other traits were below 5%. As analysis of functional diversity can be sensitive to missing values (we did not use imputation of missing values)^58^, we repeated all statistical analyses on functional diversity excluding body size and lecty and obtained highly similar results for all metrics. Taxonomy of all species was checked and standardized where necessary using the Integrated Taxonomy Information System (https://www.itis.gov/).

For studies in which the functional and/or taxonomic composition could have been biased by the sampling method (for example, sampling by trap nests excluding ground-nesting bees), biased traits were excluded from the calculation of functional diversity and the entire study was excluded from the analysis of phylogenetic diversity and MPD. For this reason, sample sizes varied slightly between analyses.

### Proportion of SNH in landscapes

The proportion of SNH in agricultural landscapes was provided by holders of primary datasets (Supplementary Fig. 3), which has the advantage that the classification of bee-relevant SNH was based on local expert knowledge. SNH categories included forests, hedgerows, extensively managed grasslands and floral enhancements under agri-environment schemes (Supplementary Table 2). Studies measured the proportion of SNH in a radius of either 0.5 km or 1 km (Supplementary Table 2), matching the scale of landscape structure considered most appropriate by dataset holders on the basis of typical foraging ranges of bees in the study systems^77^.

To examine whether landscape configuration modulates effects of the proportion of SNH on bee community properties in crop fields, we calculated edge density on the basis of the global maps of LULC from 2020 derived from ESA Sentinel-2 imagery at 10-m resolution^78^. Forest, rangeland (including natural meadows, pastures, moderate cover of bushes and shrubs) and arable land were considered the most relevant land-use classes for the question addressed in this study (how configuration of SNH may shape its capacities to buffer effects of pesticide hazard on bee communities) and were therefore included in calculations of edge densities, while the remaining classes (water, bare ground and built area) were combined into a single class. Calculations were performed within a 1-km radius (which was the radius most frequently used for the quantification of SNH) with the lsm_l_ed function from the landscapemetrics package in R (ref. ^79^). Additionally, these global land-use data were used to calculate SNH as proportion of forest and/or rangeland, confirming that direct measures of SNH from primary studies consistently correlated better with bee community descriptors than those obtained from the Sentinel-2 LULC (Supplementary Fig. 3d). We therefore used the data from primary studies for all subsequent analyses. SNH values provided by studies and those derived from Sentinel-2 LULC were fairly well correlated, with correlation coefficients (*r*) ranging from 0.12 to 1.0 and a median of 0.91. The correlation did not depend on the year in which the study was conducted, suggesting that Sentinel-2 LULC predictions vary more with regional landscape composition and structure than with past land-use changes.

### Pesticide hazard

We used two measures of field-realistic pesticide hazard for bees, depending on the data provided by each study. For 27 datasets, pesticide-use intensity was classified as either high or low depending on the local production system (conventional or organic). For this classification we only considered studies that had collected information on typical pesticide application protocols associated with the different production systems (that is, farmer interviews at a subset of sites), confirming a higher toxicity to bees in conventional compared with organic production systems. For 28 datasets, pesticide hazard was quantified as HQ based on farmers’ pesticide application records and the toxicity of pesticides to bees. To calculate HQ, we used spray records of insecticides, fungicides and herbicides as provided in primary datasets^13^ and, where available, seed treatments with neonicotinoids^7^:

$${\rm{HQ}}=\mathop{\sum }\limits_{n=1}^{{\rm{N}}}\log \frac{{\rm{application\; rate}}\left({\rm{active\; ingredient\; per\; ha}}\right)}{{{\rm{LD}}}_{50}}$$

HQ sums up all N applications of a site, considering the application rate of the active ingredient and the toxicity of systemic pesticides (oral lethal dose 50 (LD_50_) from honey bees)^80,81^. To evaluate the suitability of the HQ, corresponding HQs were calculated for oral, contact or both exposure pathways. The HQ, as defined above, was selected for further analysis as it showed the best prediction (based on correlation coefficient *r*) of bee abundance, species richness and functional and phylogenetic diversity (Supplementary Fig. 3c). Three datasets (but no study) only contained two distinct values of HQ across all sites (two datasets only included herbicide applications and one a single fungicide application at two sites). These datasets were removed from the data, since several analyses require a gradient of HQ.

Log transformation was included in the calculation of HQ to account for the nonlinear relationships of dose–response curves for individual applications^80,82^. Transformed data showed better predictions for bee community metrics obtained from the quantitative synthesis as well as for pesticide risk measured in ref. ^9^.

In total, we collected information on 6,667 pesticide applications, including 277 active ingredients, considering applications from the beginning of the season until the last date of bee sampling. The information for the concentration of active ingredients in the applied products was gathered from the national product labels (either made available by national web pages of production companies or by national pesticide databases such as the US Environmental Protection Agency). Application rates were available from primary studies based on farmer interviews and, where missing (that is, when farmers reported only the product applied without providing the application rate), were assumed to be those recommended for respective crop and development stage by the national product label. Oral LD_50_ from honey bees were obtained from the pesticide properties database and bio pesticides database^83^. For active ingredients with unbounded estimates (‘>’ that is, based on limit tests; mostly fungicides and herbicides that contribute little to pesticide hazard; 64% of active ingredients) minimal LD_50_ were used. For some active ingredients where no oral LD_50_ value could be obtained (6% of active ingredients), contact LD_50_ was used as a proxy. For single applications that only included information about pesticide type (for example, herbicides, 3% of applications), HQ was imputed as the mean HQs of the same pesticide type and production system (conventional or organic) within the same study or, if entirely missing from the study, as the mean of the same pesticide type and production system across studies.

A limitation of the HQ used here is that only pesticide use on the focal field was considered, while bees that forage in several crop fields may also be exposed to other agrochemicals^35^. For this reason, residue data obtained from bee-collected pollen have increasingly been used to quantify pesticide risk (PR) for specific bee species^9,29^, whereas this is not feasible when investigating bee communities. However, to test how well HQ predicts PR, we used the recently published dataset from ref. ^9^. Pesticide application and residue data from bumble bee pollen stores were available for 86 sites across seven countries in Europe. At each site three bumble bee colonies were placed either at a canola field or an apple orchard during the flowering period. All pesticide applications to focal fields during this period were recorded, while pesticide residues were quantified at the end of crop bloom. HQ was calculated with the formula provided above, while PR was quantified as described in ref. ^9^. These data were then analysed with a linear mixed effects model with PR as response and the HQ as explanatory variable. Country was included as random intercept and crop as covariate. Results showed that the HQ, as defined above, predicted pesticide risk fairly well, with *R*^2^ = 0.45 (*P* < 0.001) (Supplementary Fig. 3e), demonstrating the robustness of our approach. Moreover, the effects of pesticide hazard (pesticide-use intensity or HQ) on wild bee community descriptors remained consistent regardless of focal field size, further supporting the robustness of our findings across the different scales of pesticide hazard quantification (Supplementary Table 5).

Because pesticide hazard in focal fields and bee community descriptors were mostly quantified within a single year (Supplementary Table 1), it is possible that our approach primarily captures lethal and sublethal effects on adult foraging bees, as well as potential impacts on worker development in social species. Fully assessing population-level effects, including next-generation impacts, would require long-term studies. However, in the studies that assessed pesticide hazard over several years, pesticide HQ values were, on average, moderately correlated across subsequent years (*r* = 0.53), indicating that HQ measurements are generally representative of past management practices as well.

### Statistical analysis of effects of stressors on bee assemblage descriptors

Linear mixed effects models were used to test for the effects of pesticide hazard and the proportion of SNH in surrounding landscapes on descriptors of bee assemblages (abundance, species richness, functional diversity, functional MPD, functional evenness, functional specialization, phylogenetic diversity and phylogenetic MPD) in crop fields. Random intercept and slope models were fit by allowing for different relationships of predictors and response variables across datasets, as recommended^84^, to better account for heterogeneity in effects across datasets and to reduce the risk for type 1 errors compared with random intercept models. All continuous explanatory and response variables were scaled (*z*-transformation) within datasets before statistical analysis to account for differences in bee sampling protocols, pesticide recording (for example, time window) and classifications of SNH necessary for comparability^1,11,56^. To test for potential buffering of pesticide effects through a high proportion of SNH in the landscape, the original models also included the interaction term between these two variables.

In some studies, sampling was repeated across several years at the same site. To avoid pseudoreplication due to repeated measures of bees during several years in these studies compared with single-year studies, mean values across years were used for bee community descriptors and HQs. Furthermore, to avoid any bias from unbalanced designs, measures from different sampling methods (for example, timed observation and pan trapping) were averaged per site. To control for small differences in sampling effort across sites within some studies, we calculated the relative bee abundance per sample. To test for potential biases by sampling incompleteness and sampling effort on species richness, additional models were fit to species richness obtained by individual-based rarefaction and extrapolation using the iNEXT function in R^85^ returning similar results as for observed species richness (Supplementary Fig. 4).

Additional models were run to ensure that results were robust and consistent across different regional contexts and scales (that is, major global growing regions, edge density, field size and bee attractiveness of crop) as well as methodological aspects of the individual studies (that is, bee sampling method, bee sampling period, landscape radius and classification of SNH) and data inclusion criteria (threshold of sampling effort for inclusion of studies). However, none of these tests revealed evidence of bias or modulatory effects (Supplementary Tables 4–9); therefore, the final models included only pesticide hazard and SNH proportion in landscapes^86^.

As pesticide hazard was measured either as HQ or as pesticide-use intensity, two models were fit for each wild bee community descriptor. These two models are not fully independent, however, as for 45% of the total 681 sites both measures of pesticide hazard were available. Additionally, since the two models returned similar estimates for the effect of SNH (Supplementary Table 10), we report SNH effect estimates from the model including pesticide-use intensity due to its larger sample size (514 sites compared with 466 in the model with HQ).

*P* values were obtained by likelihood ratio tests and model assumptions were checked by graphical validation^86^. Where necessary, transformation of response variables was done before scaling (square root transformation was used for abundance and species richness). Models showed no spatial autocorrelation, which was tested with the TestSpatialAutocorrelation function from the DHARMa package. Also, both HQ and pesticide-use intensity showed low correlation with SNH (HQ, *r* = −0.02; pesticide-use intensity, *r* = −0.11) and all models showed low multicollinearity based on variance inflation factor (VIF) values. For linear mixed effects models, we used the glmmTMB package. To test for potential publication bias, we ran additional meta-analysis models on the different metrics of alpha diversity using the metafor package^87^ and created funnel plots, which showed no evidence of publication bias. All statistical analyses were performed in R v.4.3.2^66^.

### Beta diversity—nestedness and turnover

We quantified two different components of beta diversity—nestedness and turnover. Nestedness reflects a disassembly process characterized by pruning species from the species pool resulting in a subset of species, whereas turnover indicates a species loss accompanied by simultaneous dissimilarity in species composition resulting from the replacement of a subset of the species pool (Supplementary Fig. 1).

As a measure of nestedness, we used abundance weighted nestedness of bee assemblages based on overlap and decreasing fill (WNODF)^2,88^. WNODF was calculated separately for each dataset across comparable assemblages recorded in the same crop and during the same year(s) (Supplementary Table 1). Abundance data from different years were averaged and site-by-species assemblage matrices were ordered by increasing HQ or decreasing SNH to calculate WNODF with the nestednodf function from the vegan package^89^. For each assemblage matrix, we additionally created 999 null communities by randomly permuting the site-by-species matrix with the swap algorithm from the bipartite package^90,91^, which keeps the matrix fill and marginal totals constant. WNODF values from each dataset were *z*-transformed with means and standard deviations obtained from null models^88,92^ and then tested against a null expectation of *µ* = 0 in a one-sample *t*-test. To control for pseudoreplication of datasets from the same study, different WNODF *z*-scores obtained from the same study were averaged (actual communities and null communities) before testing. Each study, therefore, represented a data point in the *t*-test, resulting in sample sizes of 19 and 26 for the analysis of pesticide hazard and SNH proportion, respectively. To test if one stressor contributed more to nestedness than the other, we used a two-sided paired Wilcoxon test, comparing WNODF *z*-scores obtained from the same study along gradients of both increasing pesticide hazard and SNH loss.

To understand patterns of nestedness and turnover of species occurrence in bee assemblages along gradients of increasing HQ and decreasing proportion of SNH, we measured turnover and nestedness across sites of increasing anthropogenic stressors with the directional.response function from the adespatial package, developed to investigate directional community changes along environmental gradients^93^. The site (rows) by species (columns) matrices containing species occurrence data were ordered either by increasing HQ or by decreasing proportions of SNH in agricultural landscapes. As sites of equal or highly similar stressor levels were common (for example, several sites with a pesticide hazard of zero), we did not compare subsequent sites along the gradients, but rather pairs of sites shifted by three positions (for example, site 1 with site 4 and so on) were compared. The Jaccard denominator was used to obtain comparable measures, independent of species number. As this analysis requires species occurrence data (which are less sensitive to annual conditions than abundance data), we pooled data from the same study collected across different years using the same standardized sampling protocol. However, bee communities sampled in different crops or with varying sampling efforts across years were analysed separately. This approach was taken to find a good compromise between having a representative gradient of HQ and SNH across a sufficient number of sites, while also ensuring standardized sampling and comparability of communities. This resulted in 30 matrices with gradients of SNH loss and 23 with gradients of pesticide hazard.

To test if losing turnover and nestedness increased with pesticide hazard or SNH loss, values from each study were compared with gaining turnover and nestedness using a random effect meta-analysis model incorporated in the metafor package^87^. Standardized mean differences were compared using a *t*-test. Model assumptions were validated graphically for all models and to ensure robustness against outliers, Cook’s distance was checked but only showed values below 0.5. Mixed effects models, including bee attractiveness of the focal crop as moderator, were simplified on the basis of Omnibus tests, resulting in random-effects models only^94^. Total variability could be fully attributed to sampling variability (H2 = 1), while heterogeneity among true effects was estimated to be zero (I2 = 0%). Accordingly, the test for heterogeneity was non-significant for all models (all *P* > 0.90).

### Reporting summary

Further information on research design is available in the Nature Portfolio Reporting Summary linked to this article.

## Supplementary information

Supplementary Information Supplementary Tables 1–10 and Figs. 1–4.

Reporting Summary

Supplementary Data 3c Statistical source data for Supplementary Fig. 3c.

Supplementary Data 3d Statistical source data for Supplementary Fig. 3d.

Supplementary Data 3e Source data for Supplementary Fig. 3e.

## Source data

Source Data Fig. 2 Statistical source data.

Source Data Extended Data Fig. 4 Statistical source data.

## Figures and Tables

**Extended Data Table 1:**
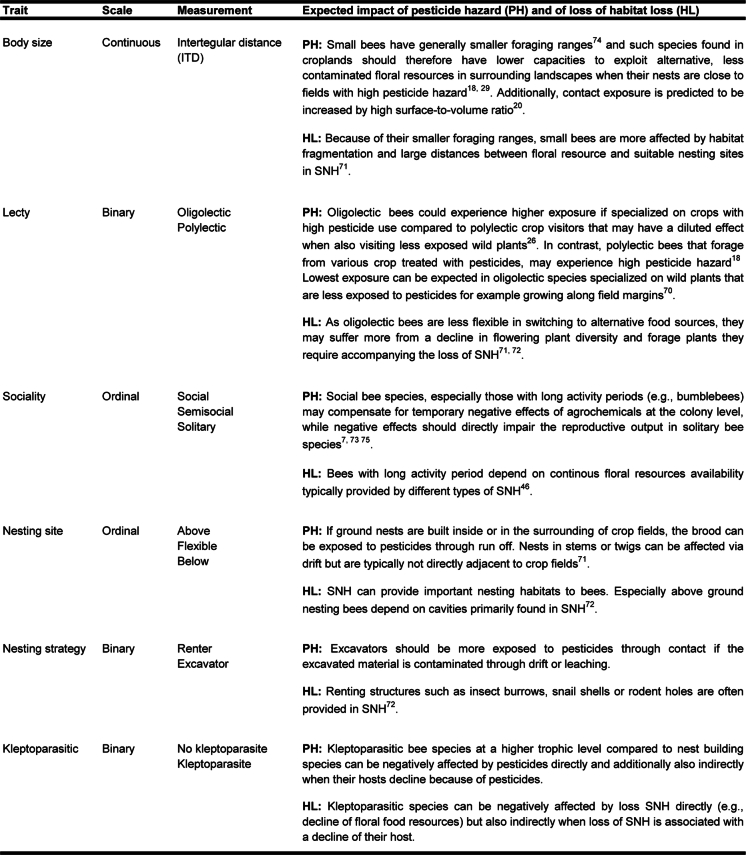
Traits expected to affect bees’ susceptibility to pesticide hazard (PH) and loss of (semi-natural) habitat (HL)

## Data Availability

The datasets generated during the study are available via FigShare at 10.6084/m9.figshare.30281617 (ref. ^95^). Source data are provided with this paper.
