# Supplementary material for: Pesticides and habitat loss additively reduce wild bees in crop fields
Source: Nat Ecol Evol. 2025 Dec 10;10(1):95–104. doi: 10.1038/s41559-025-02924-z (PMC12789018; doi:10.1038/s41559-025-02924-z)
Supplement: Supplementary file 1 — Supplementary Tables 1–10 and Figs. 1–4. [file 41559_2025_2924_MOESM1_ESM.pdf]

nature ecology & evolution

Supplementary information https://doi.org/10.1038/s41559-025-02924-z

Pesticides and habitat loss additively reduce wild bees in crop fields

In the format provided by the authors and unedited

Content

SUPPLEMENTARY TABLES **2**

Supplementary Table 1 2

Supplementary Table 2 3

Supplementary Table 3 4

Supplementary Table 4 8

Supplementary Table 5 9

Supplementary Table 6 10

Supplementary Table 7 11

Supplementary Table 8 12

Supplementary Table 9 13

Supplementary Table 10 14

SUPPLEMENTARY FIGURES **15**

Supplementary Figure 1 15

Supplementary Figure 2 16

Supplementary Figure 3 17

Supplementary Figure 4 18

SUPPLEMENTARY TABLES

Supplementary Table 1| Overview of the studies included in the quantitative synthesis.

Dataset	Study	Country	Crop type	Year(s)	Nr of sites	Reference
1<br>2<br>3	Adhikari (2019)	USA	cereals	2013<br>2014<br>2015	18	Adhikari, S. et al. (2019). Dryland organic farming partially offsets negative effects of highly simplified agricultural landscapes on forbs, bees, and bee–flower networks. Environmental Entomology, 48(4), 826-835.
4	Andersson (2013)	Sweden	cereals	2008	28	not published
5<br>6<br>7	Bushmann (2015)	USA	blueberry	2010<br>2011<br>2012	40	Bushmann, S. & Drummond, F. (2015). Abundance and diversity of wild bees (Hymenoptera: Apoidea) found in lowbush blueberry growing regions of Downeast Maine. Environmental Entomology, 44(4), 975-989.
8	Cano (2022)	Spain	olives	2018	18	Cano, D. et al. (2022). Small floral patches are resistant reservoirs of wild floral visitor insects and the pollination service in agricultural landscapes. Biological conservation, 276, 109789.
9<br>10	Carrié (2017)	France	sunflower	2013<br>2014	17	Carrié, R. et al. (2017). Interactive effects of landscape-wide intensity of farming practices and landscape complexity on wild bee diversity. Landscape Ecology, 32, 1631-1642.
11	Happe (2018)	Germany	cereal	2013	36	Happe, A. et al. (2018). Small-scale agricultural landscapes and organic management support wild bee communities of cereal field boundaries. Agriculture, Ecosystems & Environment, 254, 92-98.
12	Holzschuh (2007)	Germany	cereal	2003	42	Holzschuh, A. et al. (2007). Diversity of flower-visiting bees in cereal fields: effects of farming system, landscape composition and regional context. Journal of Applied Ecology, 44(1), 41-49.
13	Kehinde (2014)	South Africa	grape	2009	9	Kehinde, T. & Samways, M. (2014). Management defines species turnover of bees and flowering plants in vineyards. Agricultural and Forest Entomology, 16(1), 95-101.
14	Knapp (2022)	Sweden	legumes	2019	14	Knapp, J. et al. (2022). Pollinators, pests and yield—Multiple trade-offs from insecticide use in a mass-flowering crop. Journal of Applied Ecology, 59(9), 2419-2429.
15	Kovács-Hostyánszki (2011)	Hungary	cereals	2005	21	Kovács-Hostyánszki, A. et al. (2011). Local and landscape effects on bee communities of Hungarian winter cereal fields. Agricultural and Forest Entomology, 13(1), 59-66.
16<br>17	Lüscher (2014)	France	legumes<br>sunflower	2010	14	Lüscher, G. et al. (2014). Responses of plants, earthworms, spiders and bees to geographic location, agricultural management and surrounding landscape in European arable fields. Agriculture, Ecosystems & Environment, 186, 124-134.
18<br>19	Mallinger (2015)	USA	apple	2010<br>2012 - 2013	30	Mallinger, R. et al. (2015). Pesticide use within a pollinator-dependent crop has negative effects on the abundance and species richness of sweat bees, Lasioglossum spp., and on bumble bee colony growth. Journal of Insect Conservation, 19, 999-1010.
20<br>21	Marja (unpub)	Estonia	canola<br>cereals	2011	46	not published
22	Martinez-Nunez (2019)	Spain	olives	2017	39	Martínez-Núñez, C. et al. (2019). Interacting effects of landscape and management on plant–solitary bee networks in olive orchards. Functional Ecology, 33(12), 2316-2326.
23<br>24	M'Gonigell (unpub)	USA	various[1]	2006 - 2014<br>2010 - 2014	20	Bee community data published in M'Gonigle, L. K. et al. (2015). Habitat restoration promotes pollinator persistence and colonization in intensively managed agriculture. Ecological Applications, 25(6), 1557-1565.
25	Minarro (2018)	Spain	apple	2015	26	Miñarro, M. & García, D. (2018). Complementarity and redundancy in the functional niche of cider apple pollinators. Apidologie, 49, 789-802.
26	Nicholson (2017)	USA	blueberry	2013 - 2015	13	Nicholson, C. et al. (2017). Farm and landscape factors interact to affect the supply of pollination services. Agriculture, Ecosystems & Environment, 250, 113-122.
27	Otieno (2015)	Kenya	legumes	2009	12	Otieno, M. et al. (2011). Local management and landscape drivers of pollination and biological control services in a Kenyan agro-ecosystem. Biological Conservation, 144(10), 2424-2431.
28	Park (2013)	USA	apple	2009 - 2012	21	Park, M. et al. (2015). Negative effects of pesticides on wild bee communities can be buffered by landscape context. Proceedings of the Royal Society B: Biological Sciences, 282(1809), 20150299.
29	Rivers-Moore (2023)	France	cereals	2016	29	Rivers-Moore, J. et al. (2023). Contrasting effects of wooded and herbaceous semi-natural habitats on supporting wild bee diversity. Agriculture, Ecosystems & Environment, 356, 108644.
30	Rundlöf (2015)	Sweden	canola	2013	16	Rundlöf, M. et al. (2015). Seed coating with a neonicotinoid insecticide negatively affects wild bees. Nature, 521(7550), 77-80.
31	Samnegård (2019) DE	Germany	apple	2015	30	Samnegård, U. et al. (2019). Management trade-offs on ecosystem services in apple orchards across Europe: Direct and indirect effects of organic production. Journal of Applied Ecology, 56(4), 802-811.
32	Samnegård (2019) SE	Sweden	apple	2015	28	Samnegård, U. et al. (2019). Management trade-offs on ecosystem services in apple orchards across Europe: Direct and indirect effects of organic production. Journal of Applied Ecology, 56(4), 802-811.
33	Sutter (unpub)	Switzerland	apple and cherry	2018	49	not published
34	Uzmann (2020)	Germany	grape	2016	29	Uzman, D. et al. (2020). Habitat area and connectivity support cavity-nesting bees in vineyards more than organic management. Biological Conservation, 242, 108419.
35<br>36	Veromann (unpub)	Estonia	canola	2014<br>2015	36	not published

1 Bees were captured in margin hedges along crop fields and pesticide data was collected from adjacent crop fields.

Supplementary Table 2| Methods used in each study for the collection of bees, the assessment of pesticide hazard, and the classification of land-use types as semi-natural habitats (SNH) according to the categorisation by dataset developers in the primary studies.

						Consideration of habitats for SNH quantification			
Study	Bee sampling method	Sampled group	Pesticide hazard (HQ)	Pesticide use (high vs low)	Buffer radius (km)	Permanent intensive grassland	Extensive meadows, fallows and moorland	Shrubs and hedges	Forests
Adhikari (2019)	pan trap	all bees	yes	yes	1	no	yes	no	no
Andersson (2013)	pan trap & observations	all bees	no	yes	1	no	yes	no	yes
Bushmann (2015)	pan trap & observations	all bees	no	yes	1	no	yes	no	yes
Cano (unpub)	observations	all bees	no	yes	1	no	no	yes	yes
Carrié (2017)	pan trap	all bees	yes	no	0.5	no	yes	yes	yes
Happe (2018)	pan trap	all bees	yes	yes	0.5	no	yes	no	no
Holzschuh (2007)	observations	all bees	yes	yes	0.5	no	yes	yes	yes
Kehinde (2014)	pan trap	all bees	no	yes	1	no	no	yes	yes
Knapp (2022)	observations	all bees	yes	yes	1	no	yes	no	yes
Kovács-Hostyánszki (2011)	pan trap	all bees	yes	yes	0.5	no	yes	no	yes
Lüscher (2014)	observations	all bees	yes	yes	0.5	no	yes	no	yes
Mallinger (2015)	pan trap	all bees	yes	yes	1	no	yes	no	yes
Marja (unpub)	observations	*Bombus spp.*	yes	yes	1	yes	no	yes	yes
Martinez-Nunez (2019)	trap-nest	cavity-nesting bees	no	yes	1	no	yes	yes	yes
M'Gonigell (unpub)	observations	all bees	yes	yes	1	no	yes	yes	yes
Minarro (2018)	observations	all bees	yes	yes	1	no	yes	yes	yes
Nicholson (2017)	observations	all bees	no	yes	1	no	yes	yes	yes
Otieno (2015)	observations	all bees	yes	yes	1	no	no	yes	yes
Park (2013)	observations	all bees	yes	yes	1	no	yes	yes	yes
Rivers-Moore (2023)	pan trap	all bees	yes	no	0.5	yes	yes	yes	yes
Rundlöf (2015)	observations	all bees	yes	no	1	no	yes	no	yes
Samnegård (2019) DE	observations	all bees	no	yes	0.5	yes	yes	yes	yes
Samnegård (2019) SE	observations	all bees	no	yes	1	no	yes	no	yes
Sutter (unpub)	observations	all bees	yes	no	1	no	yes	yes	yes
Uzmann (2020)	trap-nest	cavity-nesting bees	yes	yes	1	no	no	yes	yes
Veromann (unpub)	pan trap	all bees	yes	no	1	no	yes	yes	yes

Supplementary Table 3| Most relevant checklist items from the PRISMA extension for ecology and evolution. Summary of how recommendations were adapted to our analytical approach of a quantitative synthesis to fulfill quality standards for data collection, analysis and reporting.

Checklist item	Sub-item	Nr.	Description by authors
Aims and questions	Provide a rationale for the review	2.1	Understanding the drivers of pollinator decline is critical, and both pesticide use and habitat loss are considered major anthropogenic threats to bee populations and the pollination services they provide. Despite growing evidence of their individual effects, the relative importance and potential combined impacts of these stressors on wild bee communities remain unclear – particularly in relation to species-specific traits such as body size and nesting strategy. This study aims to address these knowledge gaps by disentangling the individual and combined effects of pesticide exposure and habitat loss on bee assemblages across diverse agricultural landscapes.
	State the aims and scope of the review (including its generality)	2.3	At the beginning of the project, the aim of this study was defined within Work Package 7 of the POSHBEE project (https://www.poshbee.eu) as follows:<br>[Here, we aim to conduct a quantitative synthesis to identify the functional traits that define the pesticide sensitivity in bees and to explore the consequences of the environmental filter imposed by pesticides for phylogenetic and functional trait diversity in bee communities. Further, as different components of agricultural intensification can impose different environmental filters that may enhance or mitigate each other, we will also consider local and landscape factors (i.e., the amount of semi-natural habitat in agricultural landscapes) to account for collinearities and, if possible, compare the effect of the different filters. With the results of this study we aim to provide a better understanding of the risks associated with pesticide use and habitat loss.]
	State the primary questions the review addresses (e.g. which moderators were tested)	2.4	We aimed to address the following questions with this study:<br>(1) What is the relative importance of pesticide risk and habitat loss in surrounding landscapes driving wild bee abundance, species richness and functional and phylogenetic diversity in local crop fields?<br>(2) Can negative pesticide effects be buffered by a high proportion of semi-natural habitats in landscapes?<br>(3) Does pesticide use in bee-attractive crops pose a particularly high risk to bees?<br>(4) How do pesticide risk and habitat loss act as filters on bee communities selecting species with certain traits?
	Describe whether effect sizes were derived from experimental and/or observational comparisons	2.5	Effect sizes were derived from observational comparisons.
Review registration	Register review aims, hypotheses (if applicable), and methods in a time-stamped and publicly accessible archive and provide a link to the registration in the methods section of the manuscript. Ideally registration occurs before the search, but it can be done at any stage before data analysis.	3.1	Since this recommendation was published after the start of the project, no formal registration was made. However, the study aim was defined at the outset, as described in 2.3. Furthermore, the methodological approach was established as a quantitative synthesis to address the research questions outlined in 2.4.
	Describe deviations from the registered aims and methods	3.2	The original aim of the project remained unchanged, although additional research questions were defined as the project progressed, in particular how patterns of beta diversity are shaped by habitat loss and pesticide hazard.
Eligibility criteria	Report the specific criteria used for including or excluding studies when screening titles and/or abstracts, and full texts, according to the aims of the systematic review (e.g. study design, taxa, data availability)	4.1	The following inclusion criteria were used for inclusion during the screening process:<br>(1) the studies were entirely observational, with no manipulation of pesticide exposure;<br>(2) the studies characterized wild bee communities in crop fields and/or their margins;<br>(3) information on field-realistic pesticide use was collected for the focal crop field where bees were captured, or for crop fields adjacent to field margins in which bees were collected;<br>(4) the proportion of semi-natural habitat (SNH) in agricultural landscapes surrounding the local field was measured;<br>(5) the studies used a paired design with high and low pesticide use in landscapes of similar proportion of SNH, or sites were selected along independent gradients of pesticide use and proportion of SNH;<br>(6) studies identified bees to species (or morphospecies) level.
	Justify criteria, if necessary (i.e. not obvious from aims and scope)	4,2	Fulfilling the above defined criteria was necessary to address the research aim and conduct the planned statistical analysis.
Finding studies	Define the type of search (e.g. comprehensive search, representative sample)	5.1	Representative sample
	State what sources of information were sought (e.g. published and unpublished studies, personal communications)	5.2	Published and unpublished studies
	Include, for each database searched, the exact search strings used, with keyword combinations and Boolean operators	5.3	A Web of Science search (core collection database) was done with the search string "bee" AND ("wild bee abundance" OR "diversity" OR "species richness") AND ("organic" OR "production system" OR "pesticides" OR "agrochemicals" OR "insecticides" OR "fungicides"). Herbicides were not included as a search term since we aimed to study direct effects of pesticides on bees rather than indirect effects through reduced floral resource availability. Including the term “herbicides” only returned additional studies focusing on indirect effects.<br>During our literature search, we also explored Google Scholar, covering the full text of articles with the search string "species richness" AND "wild bee" AND "semi-natural" AND "pesticide" .<br>For practical reasons, the search was limited to English language.
	Provide enough information to repeat the equivalent search (if possible), including the timespan covered (start and end dates)	5.4	We included all studies published up to June 2019, which marked the final date of our literature search.
Study selection	Describe how studies were selected for inclusion at each stage of the screening process (e.g. use of decision trees, screening software)	6.1	First, the title and abstract of each report retrieved was screened for eligibility and, if not excluded based on this first screen, the full text was screened.
	Report the number of people involved and how they contributed (e.g. independent parallel screening)	6.2	Screening was done by Anina Knauer.

Data collection process	Describe where in the reports data were collected from (e.g. text or figures)	7.1	We did not extract data directly from the published reports but instead contacted the corresponding authors of suitable studies to request their raw data. To minimize potential publication bias and maximize the number of relevant datasets, we also asked them to share any additional, potentially suitable unpublished datasets, either collected by themselves or by researchers within their network.
	Describe how data were collected (e.g. software used to digitize figures, external data sources)	7.2	Corresponding authors of suitable studies were invited to complete a standardized template to share their data.
	Describe moderator variables that were constructed from collected data (e.g. number of generations calculated from years and average generation time)	7.3	Covariates were provided by the authors of the original studies using the data template (see 8.1 for a description of the data). In addition, we classified the bee attractiveness of crops (attractive: sunflower, apple, cherry, canola, legume crops; not attractive: grape, olive, cereals) and calculated pesticide hazard quotients (HQ) based on the pesticide application protocols supplied.
	State the number of extractions that were checked for accuracy by co-authors	7.6	All data collected was checked for completeness and quality (e.g. misspelled species names etc.).
Data items	Describe the key data sought from each study	8.1	The provided template included five sheets:<br>1) Data on sampled sites: coordinates, field size, crop, production system of the focal field, flower availability and species richness of flowering plants in the focal field, proportion of semi-natural habitat and arable land in the landscape, radius considered for landscape analysis<br>2) Data on pesticide applications: product, active ingredient, concentration of active ingredient in the product, applied amount per hectare, date of application<br>3) Data on sampled bees: sampling method, sampling date, bee species, abundance<br>4) Data on bee traits: bee species, inter-tegula distance (ITD), lecty, nesting site, sociality, kleptoparasitism<br>5) Land use definition: description of the habitats considered as semi-natural habitat
	Describe items that do not appear in the main results, or which could not be extracted due to insufficient information	8.2	Flower availability and species richness of flowering plants in the focal field were only recorded in a minority of the studies and could therefore not be considered in the analysis.
	Describe main assumptions or simplifications that were made (e.g. categorising both 'length' and 'mass' as 'morphology')	8.3	Bee attractiveness of crops was classified as attractive (sunflower, apple, cherry, canola, legume crops) vs. not attractive (grape, olive, cereals). Moreover, classification of semi-natural habitat varied slightly between studies as described in detail in Supplementary Table 2.
	Describe the type of replication unit (e.g. individuals, broods, study sites)	8.4	Study sites
Assessment of individual study quality	Describe whether the quality of studies included in the systematic review or meta-analysis was assessed (e.g. blinded data collection, reporting quality, experimental *versus* observational)	9.1	We verified through a survey with the corresponding authors of the original studies that the sites were selected in an unbiased and representative manner with respect to the main drivers analyzed, namely, the proportion of semi-natural habitat (SNH) and pesticide use. In all studies, sites were either selected to represent gradients in SNH proportions and/or pesticide hazard, or they were randomly selected in relation to the respective driver. Comparisons with the literature and continental databases further confirmed that the SNH gradients were representative of the respective growing regions. Thus, there is no evidence of any bias in site selection.<br>Moreover, we excluded certain datasets to maximize the reliability and robustness of our analysis and conclusions. Specifically, we removed two datasets that only included herbicide applications and one dataset with single fungicide applications at two sites. Since these datasets contained only two distinct values, an analysis of beta diversity, which requires a range of values to order sites, would not have been representative. Additionally, five more datasets were excluded due to low bee sampling effort (<10 bees per site), which could have compromised the reliability of analyses such as calculations of functional diversity metrics. These excluded studies had up to 81% missing values for certain metrics, such as functional evenness, which require a minimum number of species in the community.<br>To assess methodological heterogeneity across studies, we collected additional information on regional context and methodology, including the bee sampling method, landscape assessment radius, classification of SNH (i.e., the types of habitats considered), focal field sizes, crop type, edge density in the landscape, and sampling period.
	Describe how information about study quality was incorporated into analyses (e.g. meta-regression and/or sensitivity analysis)	9.2	We ran additional linear mixed effect models (LMMs) to test whether the reported effects of landscape composition or pesticide hazard are modulated by any of the above-described factors, but did not detect any bias due to methodological heterogeneity. A detailed description of these models can be found in the section on statistical analysis of the manuscript.<br>To ensure the robustness of our results with respect to the exclusion of studies with low sampling effort, as required for the quantification of functional diversity, we re-ran the analysis using varying exclusion thresholds.
Effect size measures	Describe effect size(s) used	10.1	For the different metrics of alpha diversity and WNODF, we used standardized effect sizes from LMMs and paired t-tests. For the meta-analysis model on turnover and nestedness, we used standardized mean differences.
Missing data	Describe any steps taken to deal with missing data during analysis (e.g. imputation, complete case, subset analysis)	11.1	We had some missing trait data for certain bee species and therefore quantified functional diversity of communities with the gawdis function in R which tolerates missing values (no imputation was performed). To ensure robustness, we ran a subset analysis excluding those traits with missing values from the calculations of functional diversity, which returned highly similar results.
	Justify the decisions made to deal with missing data	11.2	The alternative approach – imputation of missing trait values – was assessed as less conservative.
Model description	Describe the models used for synthesis of effect sizes	12.1	LMMs were used to test for the effects of pesticide hazard and the proportion of (SNH) in surrounding landscapes on descriptors of bee assemblages (abundance, species richness, functional diversity, functional MPD, functional evenness, functional specialization, phylogenetic diversity, and phylogenetic MPD) in crop fields. Random intercept and slope models were fitted, allowing for different relationships between predictors and response variables across datasets.<br>To assess patterns of beta diversity of bee communities along gradients of pesticide hazard or SNH loss, we used a random-effects meta-analysis model comparing standardized mean differences using a t-test.
Non-independence	Describe the types of non-independence encountered (e.g. phylogenetic, spatial, multiple measurements over time)	14.1	Data from the same dataset is not independent. Within a study, there is spatial non-independence across sites. Also, in studies carried out across multiple years, data from different years are not independent.
	Describe how non-independence has been handled	14.2	We handled non-independence within datasets by including random terms in LMMs and averaging data collected from different years at the same site. These models showed no spatial autocorrelation.

Meta-regression and model selection	Provide a rationale for the inclusion of moderators (covariates) that were evaluated in meta-regression models	15.1	Covariates considered in the LMMs were major study region, bee-attractiveness of crop, the bee sampling method and period, the radius of landscape assessments, landscape configuration, and the sizes of focal fields.
	Justify the number of parameters estimated in models, in relation to the number of effect sizes and studies (e.g. interaction terms were not included due to insufficient sample sizes)	15.2	We did not include any covariates in our final LMMs, as they were dropped based on likelihood ratio tests. The outputs of these tests can be found in Supplementary Tables 4–7. Final models included between 201 and 251 observations per explanatory variable.
	Describe any process of model selection	15.3	
Publication bias and sensitivity analyses	Describe assessments of the risk of bias due to missing results (e.g. publication, time-lag, and taxonomic biases)	16.1	To test for potential publication bias, we ran meta-analysis models on the different metrics of alpha and beta diversity to create funnel plots. No evidence for a publication bias was found.
	Describe any other analyses of robustness of the results, e.g. due to effect size choice, weighting or analytical model assumptions, inclusion or exclusion of subsets of the data, or the inclusion of alternative moderator variables in meta-regressions	16.3	To ensure the robustness of our results with respect to the exclusion of studies with low sampling effort (< 10 bees on average per site), we re-ran the analysis using varying exclusion thresholds as shown in Supplementary Table 9.<br>Since certain forest types may provide poor habitats for bees, we also ran all models with SNH excluding forests. However, this substantially reduced the positive effects of SNH (Supplementary Table 8), highlighting that forests were generally valuable to bees across the studied agricultural landscapes. Further, we quantified SNH based on the Sentinel-2 LULC, considering different habitat types, which also showed generally lower predictions of bee community metrics, confirming a low risk of bias related to the selection of habitats for the quantification of SNH in primary studies (Supplementary Fig. 3).
Results of study selection process	Report the number of studies screened	19.1	The search in Web of Science yielded a total of 170 publications.<br>The search in Google Scholar yielded a total of 482 publications.
	Report the number of studies excluded at each stage of screening	19.2	From the 170 studies identified in the Web of Science search 121 were excluded after the screen of abstract, and another 19 after a screen of the full text.<br>The search in Google Scholar did not yield any additional studies fulfilling inclusion criteria.<br>From the 30 studies identified as suitable, 19 agreed to share their data. Data from another 12 studies identified through the network of contacted researchers who pointed out additional suitable datasets.
	Report brief reasons for exclusion from the full text stage	19.3	Studies were excluded because of low taxonomic resolution (bee species were not identified to species or morphospecies level) or lack of landscape data.
	Present a Preferred Reporting Items for Systematic Reviews and Meta-Analyses (PRISMA)-like flowchart (www.prisma-statement.org).	19.4	A PRISMA flowchart adapted to the workflow of this quantitative synthesis can be found in Supplementary Fig. 2.
Sample sizes and study characteristics	Report the number of studies and effect sizes for data included in meta-analyses	20.1	We included 26 studies covering 36 datasets for this quantitative synthesis. We defined a dataset as data collected by the same group of researchers for a particular crop species across a replicated set of different study sites in the same time period.
	Report the number of studies and effect sizes for subsets of data included in meta-regressions	20.2	For the additional analysis testing for effects of covariates, all datasets were considered except for models testing for the modulating effects of field size, in which 6 studies without information on the size of the focal fields were excluded.
	Provide a summary of key characteristics for reported outcomes (either in text or figures; e.g. one quarter of effect sizes reported for vertebrates and the rest invertebrates)	20.3	Key characteristics of the studies and their number of sites are given in Extended Data Fig. 1.
	Provide a summary of characteristics related to individual study quality (risk of bias)	20.5	Characteristics related to study quality are given in Supplementary Table 1 and 2.

<table>
<tr>
<td>Outcomes of publication bias and sensitivity analyses</td>
<td>Provide results for the assessments of the risks of bias (e.g. Egger's regression, funnel plots)</td>
<td>24.1</td>
<td>
Funnel plots show no evidence of publication bias.

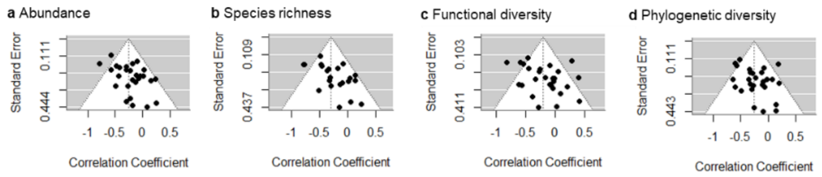

Funnel plots of meta-analysis models on the correlation coefficient between different metrics of bee communities and pesticide HQ, (a) Abundance. (b) Species richness. (c) Functional diversity. (d) Phylogenetic diversity.

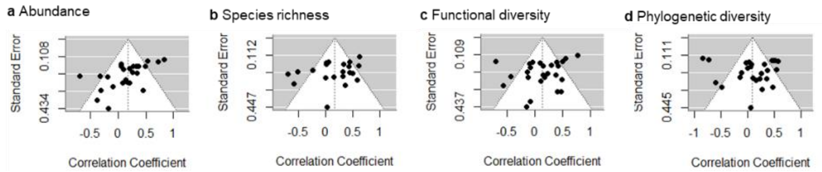

Funnel plots of meta-analysis models on the correlation coefficient between different metrics of bee communities and SNH, (a) Abundance. (b) Species richness. (c) Functional diversity. (d) Phylogenetic diversity.

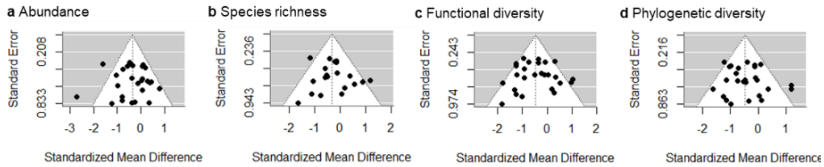

Funnel plots of meta-analysis models on the SMD between low and high pesticide use intensity, (a) Abundance. (b) Species richness. (c) Functional diversity. (d) Phylogenetic diversity.

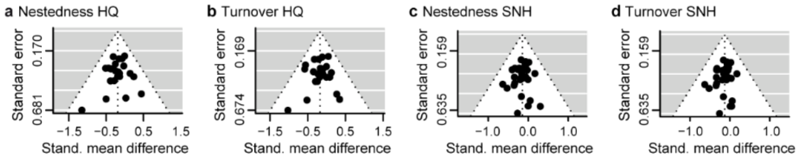

Funnel plots of meta-analysis models comparing losing against gaining components of beta-diversity, (a) Nestedness due to increasing pesticide hazard (HQ) in crop fields. (b) Turnover due to increasing HQ in crop fields. (c) Nestedness due to decreasing semi-natural habitat (SNH) in landscapes. (d) Turnover due to decreasing SNH in landscapes.
</td>
</tr>
</table>

Supplementary Table 4| Tests for the robustness of the non-buffering role of semi-natural habitats (SNH) on pesticide effects across landscape configurations. Likelihood ratio tests were conducted to examine three-way interactions between edge density and field size with pesticide hazard (pesticide-use intensity or hazard quotient (HQ)) and the proportion of SNH on key wild bee community descriptors.

Field size					
		Pesticide use intensity x SNH		HQ x SNH	
**Response**	**df**	**λ_{LRT}**	***p***	**λ_{LRT}**	***p***
Abundance	1	0.15	0.70	0.01	0.91
Species richness	1	1.42	0.23	0.10	0.75
Functional diversity	1	0.42	0.52	0.08	0.78
Phylogenetic diversity	1	1.12	0.29	0.08	0.77

Edge density					
		Pesticide use intensity x SNH		HQ x SNH	
**Response**	**df**	**λ_{LRT}**	***p***	**λ_{LRT}**	***p***
Abundance	1	0.0004	0.89	0.16	0.69
Species richness	1	0.10	0.75	0.66	0.42
Functional diversity	1	0.16	0.69	0.15	0.70
Phylogenetic diversity	1	1.21	0.27	0.08	0.77

Supplementary Table 5| Tests for the robustness of pesticide hazard effects against bee attractiveness of focal crops and different sizes of focal fields. Likelihood ratio tests were conducted to examine the interaction effects of bee attractiveness of focal crops (attractive: sunflower, apple, cherry, canola, legume crops; not attractive: grape, olive, cereals) and size of focal fields with pesticide hazard (pesticide-use intensity or hazard quotient (HQ)) on key wild bee community descriptors.

		**Bee attractiveness**			
		**Pesticide use intensity**		**HQ**	
**Response**	**df**	λ_{LRT}	***p***	λ_{LRT}	***p***
Abundance	1	2.89	0.09	1.36	0.24
Species richness	1	1.41	0.24	2.79	0.09
Functional diversity	1	2.02	0.16	2.19	0.15
Phylogenetic diversity	1	1.12	0.29	0.69	0.41
		**Field size**			
		**Pesticide use intensity**		**HQ**	
**Response**	**df**	λ_{LRT}	***p***	λ_{LRT}	***p***
Abundance	1	0.72	0.40	0.06	0.80
Species richness	1	0.34	0.56	0.55	0.46
Functional diversity	1	0.04	0.85	0.02	0.90
Phylogenetic diversity	1	0.18	0.67	1.06	0.30

Supplementary Table 6| Tests for the robustness of results against major growing regions and methodological differences across studies. Likelihood ratio tests were conducted to examine the interaction effects of major growing regions (North America, Europe, Africa), bee sampling method (pan traps vs. timed observations) and bee sampling period (time between the first and last sampling of bees) with pesticide hazard (pesticide-use intensity or hazard quotient (HQ)) and the proportion of semi-natural habitats (SNH) on key wild bee community descriptors.

**Major growing region**							
		**Pesticide use intensity**		**HQ**		**SNH**	
**Response**	**df**	λ_{LRT}	***p***	λ_{LRT}	***p***	λ_{LRT}	***p***
Abundance	2	4.62	0.10	0.62	0.73	1.87	0.39
Species richness	2	5.63	0.06	0.19	0.91	2.25	0.32
Functional diversity	2	5.70	0.06	1.06	0.59	0.67	0.72
Phylogenetic diversity	2	4.30	0.12	0.07	0.96	1.86	0.39
**Sampling method**							
		**Pesticide use intensity**		**HQ**		**SNH**	
**Response**	**df**	λ_{LRT}	***p***	λ_{LRT}	***p***	λ_{LRT}	***p***
Abundance	2	0.30	0.86	1.39	0.49	2.98	0.23
Species richness	2	0.06	0.97	0.28	0.87	4.19	0.12
Functional diversity	2	0.64	0.73	0.57	0.75	0.83	0.66
Phylogenetic diversity	2	0.06	0.97	1.17	0.56	1.83	0.40
**Sampling period**							
		**Pesticide use intensity**		**HQ**		**SNH**	
**Response**	**df**	λ_{LRT}	***p***	λ_{LRT}	***p***	λ_{LRT}	***p***
Abundance	1	0.27	0.60	0.86	0.35	0.31	0.58
Species richness	1	0.81	0.36	1.70	0.19	0.43	0.51
Functional diversity	1	0.03	0.86	0.49	0.48	0.55	0.46
Phylogenetic diversity	1	0.06	0.80	0.82	0.37	0.24	0.61

Supplementary Table 7| Tests for the robustness of semi-natural habitat (SNH) effects against different landscape radii. Likelihood ratio tests were conducted to examine the interaction effects of landscape radius (0.5 vs. 1 km) with SNH on key wild bee community descriptors.

		**Landscape radius**	
		**SNH**	
**Response**	**df**	**λ_{LRT}**	***p***
Abundance	1	0.50	0.48
Species richness	1	1.41	0.23
Functional diversity	1	1.24	0.27
Phylogenetic diversity	1	1.18	0.28

Supplementary Table 8| Effect of semi-natural habitats (SNH) on key wild bee community descriptors excluding forest as habitat.

	SNH without forest		
**Response**	**Estimate [LCL, UCL]**	χ^2	***p***
Abundance	0.06 [-0.02, 0.15]	1.98	0.16
Species richness	0.06 [-0.02, 0.15]	2.00	0.16
Functional diversity	-0.01 [-0.10, 0.08]	0.03	0.86
Phylogenetic diversity	0.04 [-0.05, 0.13]	0.85	0.36

Supplementary Table 9 | Effects of pesticide hazard (pesticide-use intensity or hazard quotient (HQ)) and semi-natural habitats (SNH) on wild bee community descriptors across models with varying exclusion criteria. Results from models applying different thresholds for excluding studies based on sampling effort (i.e., the average number of bees sampled per site). The final models applied a threshold of 10 individuals per site, which is necessary for reliably quantifying species richness and functional diversity.

	Pesticide use intensity			HQ			SNH		
**Response**	**Estimate [LCL, UCL]**	**χ^2**	***p***	**Estimate [LCL, UCL]**	**χ^2**	***p***	**Estimate [LCL, UCL]**	**χ^2**	***p***
**Threshold 0**									
Abundance	-0.30 [-0.47, -0.14]	13.31	< 0.001	-0.21 [-0.30, -0.12]	22.37	< 0.001	0.16 [0.07, 0.24]	12.53	< 0.001
Species richness	-0.34 [-0.51, -0.18]	16.65	< 0.001	-0.19 [-0.29, -0.09]	13.29	< 0.001	0.18 [0.08, 0.29]	11.24	< 0.001
Functional diversity	-0.37 [-0.54, -0.21]	19.25	< 0.001	-0.16 [-0.27, -0.05]	7.92	0.005	0.12 [0.002, 0.23]	3.95	0.047
Phylogenetic diversity	-0.34 [-0.52, -0.17]	15.15	< 0.001	-0.19 [-0.30, -0.09]	12.42	< 0.001	0.13 [0.002, 0.26]	3.99	0.046
**Threshold 10**									
Abundance	-0.30 [-0.47, -0.12]	10.94	< 0.001	-0.21 [-0.30, -0.12]	20.41	< 0.001	0.16 [0.06, 0.25]	10.51	0.001
Species richness	-0.36 [-0.53, -0.19]	17.51	< 0.001	-0.19 [-0.29, -0.09]	13.77	< 0.001	0.17 [0.06, 0.29]	8.61	0.003
Functional diversity	-0.38 [-0.55, -0.21]	19.58	< 0.001	-0.16 [-0.27, -0.05]	7.80	0.005	0.12 [-0.01, 0.24]	3.42	0.064
Phylogenetic diversity	-0.37 [-0.55, -0.19]	16.1	< 0.001	-0.21 [-0.32, -0.10]	13.73	< 0.001	0.12 [-0.02, 0.26]	2.89	0.089
**Threshold 20**									
Abundance	-0.25 [-0.45, -0.05]	6.08	0.014	-0.19 [-0.29, -0.09]	13.25	< 0.001	0.13 [0.02, 0.23]	5.49	0.019
Species richness	-0.33 [-0.51, -0.14]	12.21	< 0.001	-0.17 [-0.28, -0.06]	9.30	0.002	0.15 [0.03, 0.27]	5.72	0.017
Functional diversity	-0.35 [-0.54, -0.17]	14.20	< 0.001	-0.15 [-0.26, -0.03]	5.93	0.015	0.10 [-0.04, 0.24]	2.02	0.156
Phylogenetic diversity	-0.32 [-0.51, -0.12]	10.18	0.001	-0.19 [-0.30, -0.07]	9.89	0.001	0.11 [-0.03, 0.26]	2.26	0.133
**Threshold 40**									
Abundance	-0.25 [-0.45, -0.05]	6.08	0.014	-0.19 [-0.30, -0.08]	12.31	< 0.001	0.13 [0.02, 0.23]	5.49	0.019
Species richness	-0.33 [-0.51, -0.14]	12.21	< 0.001	-0.24 [-0.36, -0.11]	9.15	< 0.001	0.15 [0.03, 0.27]	5.72	0.017
Functional diversity	-0.35 [-0.54, -0.17]	14.20	< 0.001	-0.14 [-0.27, -0.02]	5.26	0.022	0.10 [-0.04, 0.24]	2.02	0.156
Phylogenetic diversity	-0.32 [-0.51, -0.12]	10.18	0.001	-0.19 [-0.31, -0.06]	8.71	0.003	0.11 [-0.03, 0.26]	2.26	0.133

Supplementary Table 10| Effect of semi-natural habitats (SNH) on wild bee community descriptors as obtained from the two statistical models, with pesticide hazard either measured as pesticide-use intensity or hazard quotient (HQ).

	**Pesticide use intensity + SNH**			**HQ + SNH**		
**Response**	**Estimate [LCL, UCL]**	χ^2	***p***	**Estimate [LCL, UCL]**	χ^2	***p***
Abundance	0.16 [0.062, 0.25]	10.51	**0.001**	0.19 [0.10, 0.28]	17.83	**< 0.001**
Species richness	0.17 [0.06, 0.29]	8.61	**0.003**	0.19 [0.09, 0.29]	12.94	**< 0.001**
Functional diversity	0.12 [-0.01, 0.24]	3.42	0.064	0.08 [-0.02, 0.19]	2.43	0.119
Phylogenetic diversity	0.12 [-0.02, 0.26]	2.89	0.089	0.16 [0.06, 0.26]	9.56	**0.002**
Functional MPD	0.02 [-0.01, 0.14]	0.10	0.747	-0.06 [-0.16, 0.03]	1.78	0.183
Functional evenness	0.03 [-0.07, 0.12]	0.26	0.611	-0.08 [-0.19, 0.02]	2.43	0.119
Functional specialization	0.04 [-0.07, 0.15]	0.48	0.491	0.01 [-0.10, 0.12]	0.02	0.898
Phylogenetic MPD	0.06 [-0.09, 0.21]	0.58	0.445	-0.03 [-0.17, 0.12]	0.14	0.705

SUPPLEMENTARY FIGURES

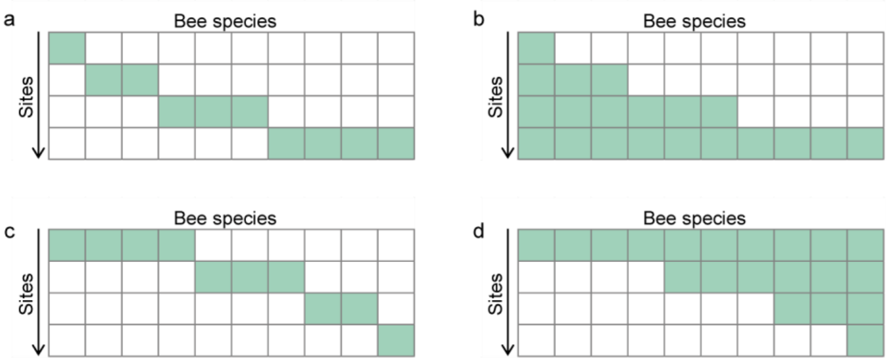

Supplementary Fig. 1 | Illustration of turnover and nestedness among bee communities along environmental gradients. a, Gaining turnover. **b**, Gaining nestedness. **c**, Loosing turnover. **d**, Loosing nestedness. Each row represents a site, and each column represents a bee species. Green-highlighted fields indicate that the species is present at the corresponding site. The arrow indicates that sites are ordered along environmental gradients.

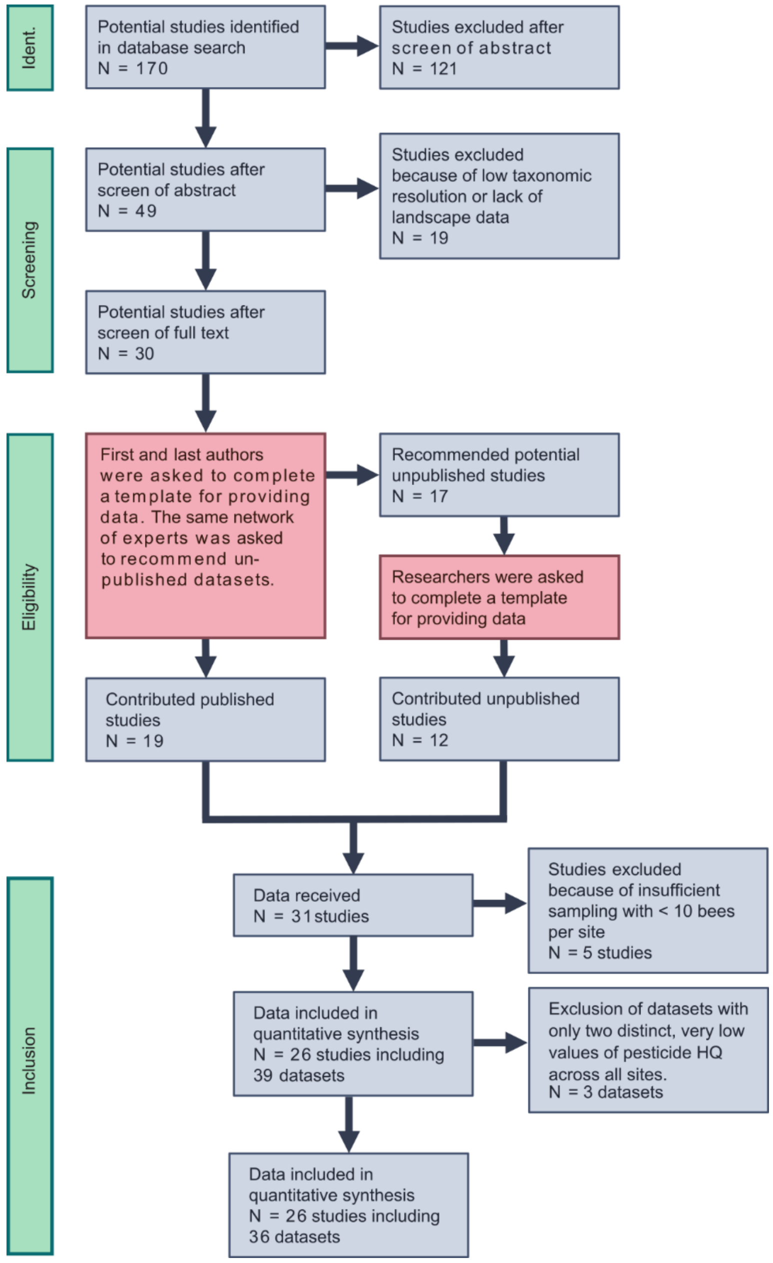

Supplementary Fig. 2 | Data used in the study. Adapted PRISMA flowchart showing each step of dataset search from the identification of literature on the topic to inclusion in the study.

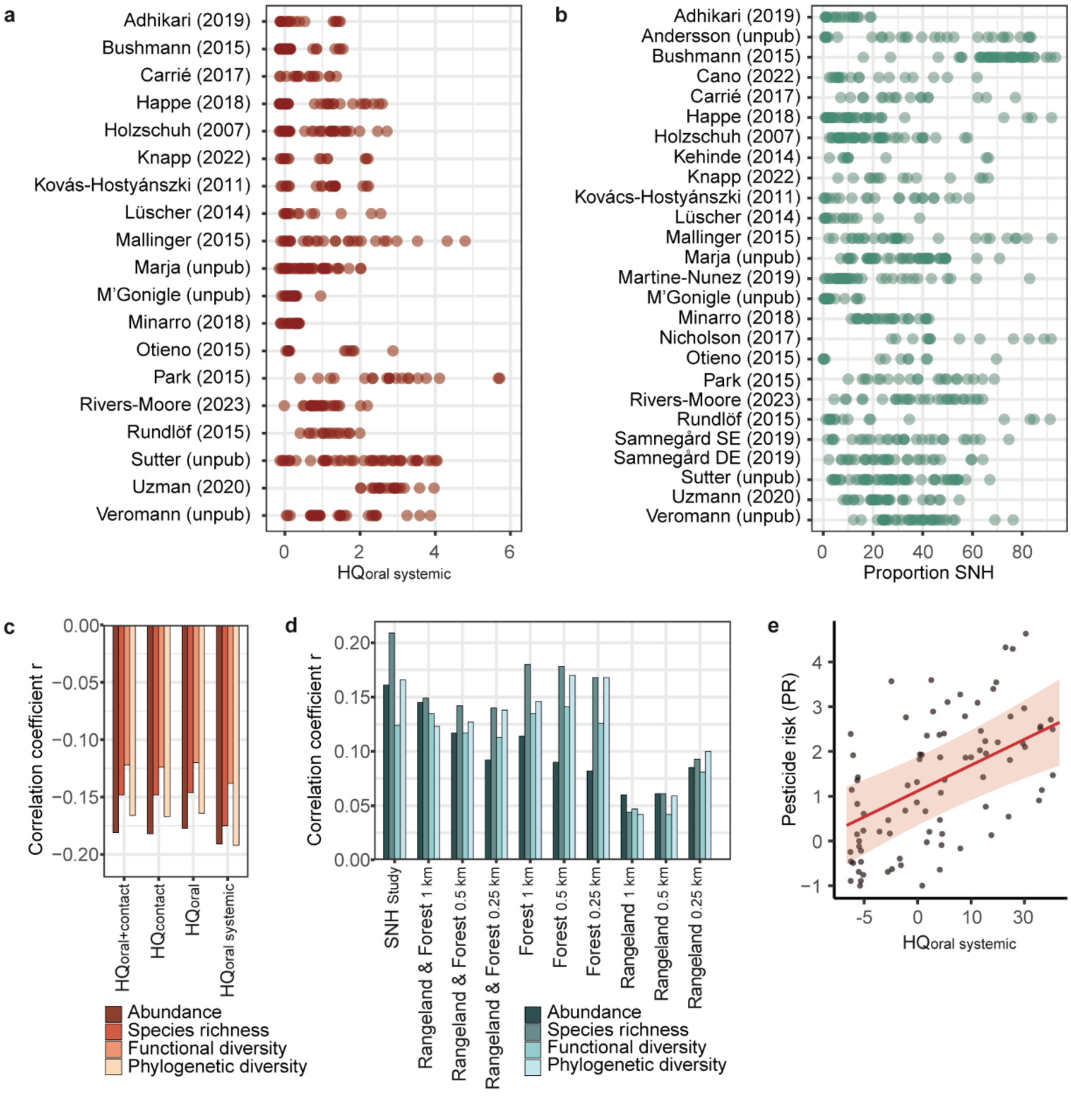

Supplementary Fig. 3 | Methodological specifications relevant for the quantification of pesticide hazard (HQ) and proportion of semi-natural habitat (SNH) in agricultural landscapes. a, Distribution of HQ values within and across study regions ($HQ_{oral\ systemic}$; showing highest correlation with bee community descriptors and therefore used in the analysis). Shown are mean values per week and crop field to account for differences in sampling periods across studies (N = 19). **b**, Distribution of SNH percentages within and across study regions (N = 20). **c**, Correlations (based on Pearson correlation coefficients, r) of different hazard quotients (HQs) with bee community descriptors. Different HQs either considered the pesticides' oral or contact LD_{50} values from honeybees (*Apis mellifera*) or the sum of these. The $HQ_{oral\ systemic}$ only considered systemic pesticides (47% of applications), which have a higher likelihood of resulting in oral exposure (N = 681 sites). **d**, Correlations of the proportion of SNH as provided by primary datasets (SNH_{study}) or estimated as the proportion of forest or rangeland and forest based on the Sentinel-2 global map of land use/land cover (LULC) on a radius of 0.25, 0.5 or 1 km with bee community descriptors (N = 681 sites). **e**, Relationship between pesticide hazard quotient HQ calculated from pesticide applications to the focal crop field and pesticide risk PR from pesticide residue data obtained from bee collected pollen (N = 86 sites across 7 countries in Europe). Shown is the estimate ± 95% CI derived from a two-sided linear mixed effects model accounting for non-independence within study regions (countries). HQ predicted PR well with R^2 = 0.45 ($p < 0.001$).

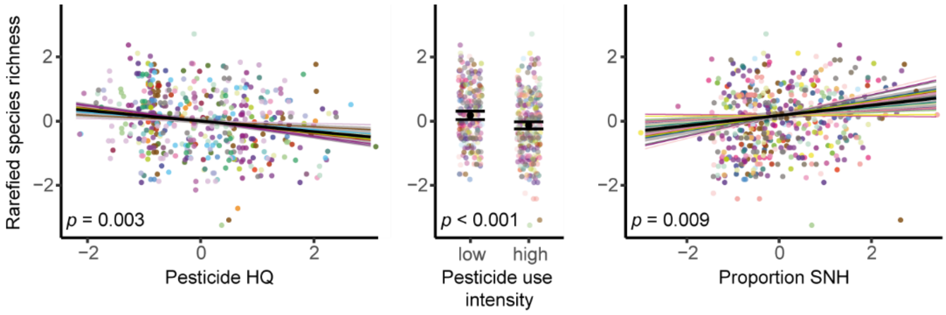

Supplementary Fig. 4 | Effects of pesticide hazard and semi-natural habitat (SNH) loss on rarefied species richness corroborate the effects on observed species richness. Estimates ± 95% CI derived from two-sided linear mixed effects models accounting for non-independence within dataset (N = 681 sites) on the effect of the pesticide hazard quotient (HQ, calculated from pesticide application protocols considering application rates and the toxicity of active ingredients to bees) (left), high pesticide-use intensity (based on production system considering typical application protocols) (middle) and the proportion of SNH (right) in surrounding landscapes. Colors indicate datasets and corresponding random slopes.
