# Supplementary material for: Pesticides and habitat loss additively reduce wild bees in crop fields
Source: Nat Ecol Evol. 2025 Dec 10;10(1):95–104. doi: 10.1038/s41559-025-02924-z (PMC12789018; doi:10.1038/s41559-025-02924-z)
Supplement: Supplementary file 2 — Reporting Summary [file 41559_2025_2924_MOESM2_ESM.pdf]

nature portfolio

Corresponding author(s): Anina Knauer

Last updated by author(s): Oct 5, 2025

Reporting Summary

Nature Portfolio wishes to improve the reproducibility of the work that we publish. This form provides structure for consistency and transparency in reporting. For further information on Nature Portfolio policies, see our Editorial Policies and the Editorial Policy Checklist.

Statistics

For all statistical analyses, confirm that the following items are present in the figure legend, table legend, main text, or Methods section.

n/a	Confirmed	
☐	☒	The exact sample size (*n*) for each experimental group/condition, given as a discrete number and unit of measurement
☐	☒	A statement on whether measurements were taken from distinct samples or whether the same sample was measured repeatedly
☐	☒	The statistical test(s) used AND whether they are one- or two-sided<br>*Only common tests should be described solely by name; describe more complex techniques in the Methods section.*
☐	☒	A description of all covariates tested
☐	☒	A description of any assumptions or corrections, such as tests of normality and adjustment for multiple comparisons
☐	☒	A full description of the statistical parameters including central tendency (e.g. means) or other basic estimates (e.g. regression coefficient) AND variation (e.g. standard deviation) or associated estimates of uncertainty (e.g. confidence intervals)
☐	☒	For null hypothesis testing, the test statistic (e.g. *F*, *t*, *r*) with confidence intervals, effect sizes, degrees of freedom and *P* value noted<br>*Give P values as exact values whenever suitable.*
☒	☐	For Bayesian analysis, information on the choice of priors and Markov chain Monte Carlo settings
☒	☐	For hierarchical and complex designs, identification of the appropriate level for tests and full reporting of outcomes
☐	☒	Estimates of effect sizes (e.g. Cohen's *d*, Pearson's *r*), indicating how they were calculated

Our web collection on statistics for biologists contains articles on many of the points above.

Software and code

Policy information about availability of computer code

Data collection	ArcGIS Pro version 3.2.2. was used to process land use/land cover (LULC) data derived from ESA Sentinel-2 imagery.
Data analysis	All data analysis was done with R version 4.3.2 using the following packages:<br>- mFD<br>- BAT<br>- Picante<br>- gawdis<br>- landscapemetrics<br>- metafor<br>- vegan<br>- bipartite<br>- adespatial<br>- glmmTMB<br>- DHARMa

For manuscripts utilizing custom algorithms or software that are central to the research but not yet described in published literature, software must be made available to editors and reviewers. We strongly encourage code deposition in a community repository (e.g. GitHub). See the Nature Portfolio guidelines for submitting code & software for further information.

Data

Policy information about availability of data

All manuscripts must include a data availability statement. This statement should provide the following information, where applicable:

- Accession codes, unique identifiers, or web links for publicly available datasets
- A description of any restrictions on data availability
- For clinical datasets or third party data, please ensure that the statement adheres to our policy

The datasets generated during the current study will be available through figshare: https://figshare.com/s/7472da16cbcb250b2036

Research involving human participants, their data, or biological material

Policy information about studies with human participants or human data. See also policy information about sex, gender (identity/presentation), and sexual orientation and race, ethnicity and racism.

Reporting on sex and gender	*Use the terms sex (biological attribute) and gender (shaped by social and cultural circumstances) carefully in order to avoid confusing both terms. Indicate if findings apply to only one sex or gender; describe whether sex and gender were considered in study design; whether sex and/or gender was determined based on self-reporting or assigned and methods used. Provide in the source data disaggregated sex and gender data, where this information has been collected, and if consent has been obtained for sharing of individual-level data; provide overall numbers in this Reporting Summary. Please state if this information has not been collected. Report sex- and gender-based analyses where performed, justify reasons for lack of sex- and gender-based analysis.*
Reporting on race, ethnicity, or other socially relevant groupings	*Please specify the socially constructed or socially relevant categorization variable(s) used in your manuscript and explain why they were used. Please note that such variables should not be used as proxies for other socially constructed/relevant variables (for example, race or ethnicity should not be used as a proxy for socioeconomic status). Provide clear definitions of the relevant terms used, how they were provided (by the participants/respondents, the researchers, or third parties), and the method(s) used to classify people into the different categories (e.g. self-report, census or administrative data, social media data, etc.) Please provide details about how you controlled for confounding variables in your analyses.*
Population characteristics	*Describe the covariate-relevant population characteristics of the human research participants (e.g. age, genotypic information, past and current diagnosis and treatment categories). If you filled out the behavioural & social sciences study design questions and have nothing to add here, write "See above."*
Recruitment	*Describe how participants were recruited. Outline any potential self-selection bias or other biases that may be present and how these are likely to impact results.*
Ethics oversight	*Identify the organization(s) that approved the study protocol.*

Note that full information on the approval of the study protocol must also be provided in the manuscript.

Field-specific reporting

Please select the one below that is the best fit for your research. If you are not sure, read the appropriate sections before making your selection.

☐ Life sciences ☐ Behavioural & social sciences ☒ Ecological, evolutionary & environmental sciences

For a reference copy of the document with all sections, see nature.com/documents/nr-reporting-summary-flat.pdf

Ecological, evolutionary & environmental sciences study design

All studies must disclose on these points even when the disclosure is negative.

Study description	We conducted a global quantitative synthesis analysis of 36 primary datasets of published field studies on the effects of pesticide use and the availability of semi-natural habitat on wild bee assemblages in crop fields.
Research sample	Datasets were selected based on a systematic literature search of published field studies on the effects of pesticide use and the availablity semi-natural habitats on wild bee assemblages in crop fields.
Sampling strategy	The data includes all datasets shared by authors of the primary studies.
Data collection	A systematic Web of Science search (core collection database) using "bee" AND ("wild bee abundance" OR "diversity" OR "species richness") AND ("organic" OR "production system" OR "pesticides" OR "agrochemicals" OR "insecticides" OR "fungicides") was performed in June 2019. This yielded a total of 170 publications, which were checked for eligibility based on the following criteria: (1) the studies were entirely observational, with no manipulation of pesticide exposure; (2) the studies characterized wild bee communities in crop fields and/or their margins; (3) information on field-realistic pesticide use was collected for the focal crop field where bees were captured, or for crop fields adjacent to field margins in which bees were collected; (4) the proportion of semi-

	natural habitat (SNH) in agricultural landscapes surrounding the local field was measured; (5) the studies used a paired design with high and low pesticide use in landscapes of similar proportion of SNH, or sites were selected along independent gradients of pesticide use and proportion of SNH; and (6) studies identified bees to species (or morphospecies) level and had a sufficient sampling effort (> 10 bees on average per sampling site) as required for the quantification of species richness and functional diversity. Correstponding authors of suitable studies were asked to share their data and, to minimize potential publication bias and to maximize the number of relevant data sets, we asked them for further potentially suitable unpublished datasets collected by themselves or researchers in their network
Timing and spatial scale	Our quantitative synthesis covers data of 36 primary studies and data collected beteen 2003 and 2018 in a total of 681 fields across three continents (North America, Europe, Africa).
Data exclusions	Five studies were excluded from the analysis because bees were not identified to species level or because of an insufficient sampling of bee with less than 10 individuals per site on average.
Reproducibility	The methodology and statistical data analyses used are described in sufficient detail in the manuscript to ensure that they are reproducible by other researchers.
Randomization	Studies selected sites to achieve representative gradients of SNH proportions in landscapes and/or pesticide hazard, or site selection was random in relation to the respective driver.
Blinding	not applicable

Did the study involve field work? ☐ Yes ☒ No

Reporting for specific materials, systems and methods

We require information from authors about some types of materials, experimental systems and methods used in many studies. Here, indicate whether each material, system or method listed is relevant to your study. If you are not sure if a list item applies to your research, read the appropriate section before selecting a response.

Materials & experimental systems

n/a	Involved in the study
☒	☐ Antibodies
☒	☐ Eukaryotic cell lines
☒	☐ Palaeontology and archaeology
☒	☐ Animals and other organisms
☒	☐ Clinical data
☒	☐ Dual use research of concern
☒	☐ Plants

Methods

n/a	Involved in the study
☒	☐ ChIP-seq
☒	☐ Flow cytometry
☒	☐ MRI-based neuroimaging

Plants

Seed stocks	*Report on the source of all seed stocks or other plant material used. If applicable, state the seed stock centre and catalogue number. If plant specimens were collected from the field, describe the collection location, date and sampling procedures.*
Novel plant genotypes	*Describe the methods by which all novel plant genotypes were produced. This includes those generated by transgenic approaches, gene editing, chemical/radiation-based mutagenesis and hybridization. For transgenic lines, describe the transformation method, the number of independent lines analyzed and the generation upon which experiments were performed. For gene-edited lines, describe the editor used, the endogenous sequence targeted for editing, the targeting guide RNA sequence (if applicable) and how the editor was applied.*
Authentication	*Describe any authentication procedures for each seed stock used or novel genotype generated. Describe any experiments used to assess the effect of a mutation and, where applicable, how potential secondary effects (e.g. second site T-DNA insertions, mosiacism, off-target gene editing) were examined.*
